# Comparative In Vitro Killing of Key Bovine Respiratory Bacterial Pathogens by Pradofloxacin and Eight Other Veterinary Antimicrobial Agents

**DOI:** 10.3390/microorganisms13122681

**Published:** 2025-11-25

**Authors:** Joseph M. Blondeau, Shantelle D. Fitch

**Affiliations:** 1 Departments of Biochemistry, Microbiology and Immunology, Pathology and Laboratory Medicine and Ophthalmology, University of Saskatchewan, Saskatoon, SK S7N 0W8, Canada; 2 Department of Clinical Microbiology, Royal University Hospital and Saskatchewan Health Authority, Saskatoon, SK S7N 0W8, Canada; shantelle.fitch@saskhealthauthority.ca

**Keywords:** pradofloxacin, *P. multocida*, *M. haemolytica*, in vitro kill, bovine respiratory disease (BRD)

## Abstract

Pradofloxacin is a third-generation dual enzyme targeting bactericidal veterinary fluoroquinolone, recently approved for use in cattle for bovine respiratory disease, which is active against Gram-positive/negative, atypical and anaerobic bacteria. We compared in vitro killing by pradofloxacin to that by ceftiofur, danofloxacin, enrofloxacin, florfenicol, marbofloxacin, tildipirosin, tilmicosin and tulathromycin against bovine isolates of *Mannheimia haemolytica* and *Pasteurella multocida* over a range of bacterial densities (10^6^–10^9^ cfu/mL). Drug concentrations used in the kill assays included the minimum inhibitory and mutant prevention drug concentrations and maximum serum and maximum tissue drug concentrations. Regardless of bacteria density tested and drug concentration used, pradofloxacin consistently killed as many or more (but not fewer) bacterial cells than any other drug tested against *M. haemolytica* strains. At the 10^8^–10^9^ cfu/mL densities, pradofloxacin killed 99–99.9%, 100% and 100% of bacterial cells at the MPC, maximum serum and maximum tissue drug concentrations, respectively, following 24 h of drug exposure. Indeed, pradofloxacin killed 99.9–99.99% of cells following 30–60 min of exposure to the maximum serum concentration. Similar trends were seen with killing of *P. multocida* strains by pradofloxacin. Against high-density bacterial populations, pradofloxacin was rapidly bactericidal and consistently killed more cells than the other agents tested. This manuscript represents the most comprehensive comparative in vitro kill study completed to date.

## 1. Introduction

Bacterial killing is essential for recovery from infection. Antimicrobials remain the cornerstone for infectious disease therapy and impact morbidity and mortality [1]. Time to initiation of effective antimicrobial therapy in humans with serious infection has been shown to impact mortality [2]. Additionally, effective short-course antimicrobial therapy has been shown to be non-inferior to longer courses of therapy in human patients with mild to moderate bacterial respiratory tract infections and urinary tract infections—including pyelonephritis and others [3]. Shorter durations of therapy may reduce selective pressures for emergence of antimicrobial-resistant organisms [4].

*Mannheimia haemolytica* and *Pasteurella multocida* are primary bacterial pathogens in bovine respiratory disease (BRD)—a complex, multifactorial process where bacterial infection follows viral infection and is influenced by environmental, nutritional and management variables [5]. Timsit et al. reported that *P. multocida* (54.8%) was the most frequent bacteria recovered from cattle with BRD, followed by *M. haemolytica* (30.5%) [6]. Klima et al. reported that *M. haemolytica* was the most frequently recovered bacterial pathogen from BRD mortalities [7]. As such, investigating these two pathogens against a variety of antimicrobial agents used for treating BRD seems paramount.

Antimicrobial agents are evaluated in vitro and in vivo to determine antimicrobial activity and clinical effect, respectively. Three in vitro measurements for determining antibacterial activity include the minimum inhibitory concentration (MIC), mutant prevention concentration (MPC) and bacterial killing to differentiate antimicrobial agents that are bactericidal from those that are bacteriostatic. Additionally, in vitro kill measurements determine the speed and extent of bacterial killing over ranges of bacterial densities. Bacterial densities are not constant during infection [8], and neither are drug concentrations. For MIC testing, a standardized bacterial inoculum of 10^5^ colony forming units per milliliter (cfu/mL) are exposed to doubling dilutions of drug in broth, whereas for MPC testing, ≥10^9^ cfu are utilized against doubling drug concentrations on drug-containing agar plates. The MPC measurement is used to determine the lowest drug concentration blocking the growth of spontaneously occurring resistant subpopulations present in high-density bacterial inocula [9]. High-density bacterial populations have been reported from humans with central nervous system infections, pulmonary infections and urinary tract infections and in cattle from experimentally induced lower respiratory infections [10,11].

Pradofloxacin is a veterinary fluoroquinolone previously approved for use in companion animals [12] and most recently in cattle and swine. It is a simultaneously dual-targeting compound inhibiting both DNA gyrase (topoisomerase II) and topoisomerase IV in both Gram-positive and Gram-negative bacteria. Both enzymes are critical for DNA replication, and inhibition of these enzymes has a lethal effect. As such, pradofloxacin—like other fluoroquinolones—is bactericidal based on the traditional definition of a >3 log_10_ kill in bacterial cells [13]. As a dual-targeting drug, the emergence of resistance is argued to be rare, as an organism would need to simultaneously have two resistance mutations for growth in the presence of the drug [14]. Fluoroquinolones are not considered first-line antimicrobial agents but rather critical antibiotics with broad impact on the gut microbiome [15].

We wanted to determine the rate and extent of bacterial killing by pradofloxacin and the comparator antibiotics against high-density bacterial populations (10^6^–10^9^ cfu/mL). Such data has not been previously published for many of these agents. In a previous recent study from our laboratory, we showed rapid killing by pradofloxacin in a 180 min kill assay and against 10^5^ cfu/mL strains of either *M. haemolytica* or *Pasteurella multocida* [16]. In this study, we compared the in vitro activity (MIC, MPC, kill measurements) of pradofloxacin to that of ceftiofur, danofloxacin, enrofloxacin, florfenicol, marbofloxacin, tildipirosin, tilmicosin and tulathromycin against bovine isolates of *M. haemolytica* and *P. multocida*. Specifically, we compared killing by pradofloxacin to that of the other agents tested using the measured MIC and MPC values along with the maximum serum and maximum tissue drug concentrations and over bacterial densities ranging from 10^6^ to 10^9^ cfu/mL—bacterial densities associated with infection.

## 2. Materials and Methods

### 2.1. Bacterial Strains

Wildtype field strains of *M. haemolytica* (n = 3) and *P. multocida* (n = 3) collected from clinical trials were used. Bacteria were identified using matrix-assisted laser desorption ionization–time of flight (MALDI-TOF) and/or Vitek II (BioMerieux, St. Laurent, QC, Canada). Isolates were grown on blood agar (tryptic soy agar containing 5% sheep red blood cells [BA]) (Oxoid, Nepean, ON, Canada) in room air at 35–37 °C for 18–24 h. Isolated single colonies were sterilely transferred to skim milk in 1.2 mL cryovials and then frozen at −70 °C. All included strains were susceptible by LMIC breakpoints to each drug used in this study. Each selected isolate included in this study had to be susceptible to all antimicrobials based on currently available recommended susceptibility MIC breakpoints [17].

### 2.2. Antimicrobial Compounds

Enrofloxacin and pradofloxacin were obtained from Bayer Animal Health (Elanco as of 2020, Greenfield, IN, USA) and prepared as per the manufacturers’ instructions. Ceftiofur, tilmicosin and tulathromycin (Zoetis, Kirkland, QC, Canada), florfenicol and tildipirosin (Merck, Kirkland, QC, Canada) and marbofloxacin (Vetoquinol, Laval Trie, QC, Canada) were purchased commercially and prepared in accordance with the manufacturer’s directions. Fresh stock solutions stored at −70 °C were used for each experiment.

### 2.3. MIC Testing

For MIC testing, the procedure recommended by the Clinical and Laboratory Standards Institute was followed [18]. Strains were thawed and then sub-cultured on BA twice with each incubation for 18–24 h at 35–37 °C in O_2_. Two-fold drug concentration increments was added to 96-well micro-dilution trays (Thermo Fisher Scientific, Mississauga, ON, Canada) in Mueller–Hinton Broth (MHB) (Difco Laboratories, Detroit, MI, USA). Suspensions of *M. haemolytica* and *P. multocida* (0.5 McFarland standard) were diluted (final inoculum of 5 × 10^5^ cfu/mL), added to microtiter trays and incubated (18–24 h at 35–37 °C in O_2_). The lowest antimicrobial drug concentration preventing visible bacterial growth was recorded as the MIC. American Type Culture Collection (ATCC) control *Escherichia coli* 25922, *Enterococcus faecalis* 29212, *Staphylococcus aureus* 29213 and *Pseudomonas aeruginosa* 27853 strains were tested with each MIC assay to ensure quality control performance ranges.

### 2.4. MPC Testing

To produce a lawn of confluent growth, 5 BA plates/strain were inoculated and then incubated at 35–37 °C for 18–24 h, after which the complete plate contents were transferred to MHB (100 mL) and incubated as described [19,20]. Bacterial densities of ≥3 × 10^9^ cfu/mL were estimated from spectrophotometric (600 Dnm) readings of ≥0.3 and colony counts. After being transferred to drug-containing agar plates, ≥10^9^ (in 100 µL) were inoculated and spread. A range of tested drug concentrations from 1 dilution below the MIC to the determined MPC were used. Plates containing drug were used within 7 days. Following incubation as described, inoculated plates were screened for organism growth after 24 and 48 h. The lowest drug concentration preventing growth (48 h) was the MPC. The aforementioned 4 ATCC control strains were included in each MPC experiment.

### 2.5. Kill Experiments

Methods for kill experiments were as previously described [21]. Following incubation on BA plates (18–24 h at 35–37 °C) in room air, *M. haemolytica* and *P. multocida* strains had an inoculum transferred to MHB and were incubated (35–37 °C in O_2_ for 2 h), after which spectrophotometric readings of ≥0.3 verified cell densities ≥10^9^ cells/mL [19]. We adjusted inocula to achieve cell densities of 10^6^–10^9^ cfu/mL in MHB; to this, antimicrobial agent was added: ceftiofur, enrofloxacin, florfenicol, marbofloxacin, pradofloxacin, tildipirosin, tilmicosin or tulathromycin was added in amounts based on the measured MIC and MPC or pre-established maximum serum or tissue concentration for each drug [11]. Three aliquots of each resulting suspension were applied to drug-free BA plates with an L-shaped cell spreader (Thermo Fisher Scientific, Mississauga, ON, Canada) and incubated as described. Measurements of the degree of antimicrobial-mediated-killing log_10_ reduction in viable cells and percentage of cells killed were obtained at time 0, 30 min, 1, 2, 4, 6, 12 and 24 h following exposure to each antimicrobial agent. For each of the 3 aliquots, means of the results were calculated. Each time point represents the average of 9 individual measurements (3 aliquots × 3 individual isolates).

### 2.6. Statistical Analysis

Statistical analysis of the data was performed by means of a repeated-measures ANCOVA for each antimicrobial/log-exposure dataset, with fixed effects (drug, exposure time and drug-by-time interaction). For each model, CFU count at time 0 was included as a covariate, and a compound symmetric covariance structure was used. CFU counts were logarithmically transformed to achieve a normal distribution. Bonferroni adjustments for multiple comparisons were made. Values of *p* ≤ 0.05 were considered significant for all analyses.

## 3. Results

The MIC and MPC values for each strain tested in this study are included in Table 1 along with the maximum serum and tissue drug concentrations for each drug.

### 3.1. Mannheimia haemolytica

#### 3.1.1. Exposure to Minimum Inhibitory Drug Concentration (MIC)

**Figure 1:**
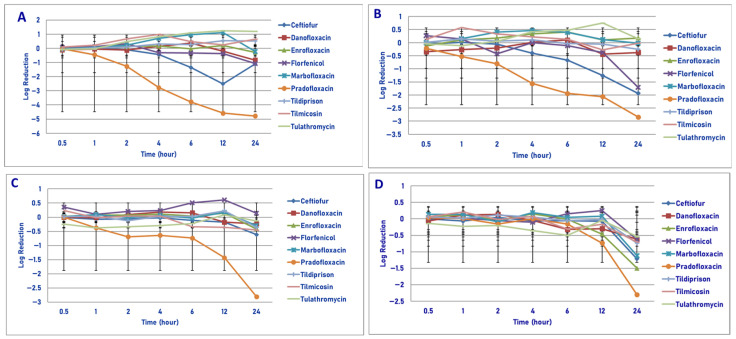
Comparative killing of *Mannheimia haemolytica* by 9 antimicrobial agents at the minimum inhibitory concentration (MIC): (**A**) 10^6^ cfu/mL, (**B**) 10^7^cfu/mL, (**C**) 10^8^ cfu/mL, (**D**) 10^9^ cfu/mL drug concentrations. (**A**) MIC 10^6^ cfu/mL. Four hours: pradofloxacin vs. marbofloxacin (*p* = 0.0128), tilmicosin (*p* = 0.0020), tulathromycin (*p* = 0.0050). Six hours: pradofloxacin vs. danofloxacin (*p* < 0.0001), enrofloxacin (*p* = 0.0004), florfenicol (*p* = 0.0161), marbofloxacin (*p* < 0.0001), tildipirosin (*p* = 0.0020), tilmicosin (*p* = 0.0001), tulathromycin (*p* < 0.0001). Twelve hours: ceftiofur vs. marbofloxacin (*p* = 0.0087), tulathromycin (*p* = 0.0022); pradofloxacin vs. danofloxacin (*p* < 0.0001), enrofloxacin (*p* < 0.0001), florfenicol (*p* = 0.0003), marbofloxacin (*p* < 0.0001), tildipirosin (*p* < 0.0001), tilmicosin (*p* < 0.0001), tulathromycin (*p* < 0.0001). Twenty-four hours: pradofloxacin vs. danofloxacin (*p* = 0.0175), enrofloxacin (*p* = 0.0016), marbofloxacin (*p* < 0.0001), tildipirosin (*p* < 0.0001), tulathromycin (*p* < 0.0001). (**B**) MIC 10^7^ cfu/mL. Six hours: pradofloxacin vs. tulathromycin (*p* = 0.0468). Twelve hours: pradofloxacin vs. tulathromycin (*p* = 0.0028). Twenty-four hours: pradofloxacin vs. enrofloxacin (*p* = 0.0006), marbofloxacin (*p* = 0.0028), tilmicosin (*p* = 0.0027), tulathromycin (*p* = 0.0007). (**C**) MIC 10^8^ cfu/mL. Twelve hours: pradofloxacin vs. enrofloxacin (*p* = 0.0373), florfenicol (*p* = 0.0328), marbofloxacin (*p* = 0.0191), tildipirosin (*p* = 0.0158), tulathromycin (*p* = 0.0501). Twenty-four hours: pradofloxacin vs. danofloxacin (*p* < 0.0001), ceftiofur (*p* < 0.0001), enrofloxacin (*p* < 0.0001), florfenicol (*p* < 0.0001), marbofloxacin (*p* < 0.0001), tildipirosin (*p* < 0.0001), tilmicosin (*p* < 0.0001), tulathromycin (*p* < 0.0001). (**D**) MIC 10^9^ cfu/mL. Twenty-four hour: pradofloxacin vs. danofloxacin (*p* < 0.0001), florfenicol (*p* = 0.0385), tildipirosin (*p* = 0.0003), tilmicosin (*p* = 0.0125), tulathromycin (*p* = 0.0133).

**Figure 2:**
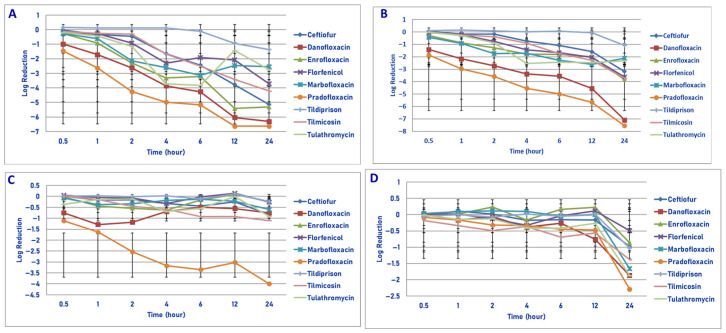
Comparative killing of *Mannheimia haemolytica* by 9 antimicrobial agents at the MPC: (**A**) 10^6^ cfu/mL, (**B**) 10^7^ cfu/mL, (**C**) 10^8^ cfu/mL, (**D**) 10^9^ cfu/mL drug concentrations. (**A**) MPC 10^6^ cfu/mL. Two hours: pradofloxacin vs. tildipirosin (*p* = 0.0242). Four hours: danofloxacin vs. tildipirosin (*p* = 0.0441); pradofloxacin vs. tildipirosin (*p* = 0.0011). Six hours: danofloxacin vs. tildipirosin (*p* = 0.0245); pradofloxacin vs. tildipirosin (*p* = 0.0015). Twelve hours: danofloxacin vs. tildipirosin (*p* = 0.0005) and tulathromycin (*p* = 0.0040), enrofloxacin vs. tildipirosin (*p* = 0.0154); pradofloxacin vs. florfenicol (*p* = 0.0143), tildipirosin (*p* < 0.0001), tulathromycin (*p* = 0.0007). Twenty-four hours: danofloxacin vs. tildipirosin (*p* = 0.0010); pradofloxacin vs. tildipirosin (*p* = 0.0006). (**B**) MPC 10^7^ cfu/mL. Two hours: pradofloxacin vs. ceftiofur (*p* = 0.0236), tildipirosin (*p* = 0.0125). Four hours: danofloxacin vs. tildipirosin (*p* = 0.0222); pradofloxacin vs. ceftiofur (*p* = 0.0029), tildipirosin (*p* < 0.0001), tilmicosin (*p* = 0.0071). Six hours: danofloxacin vs. tildipirosin (*p* = 0.0354); pradofloxacin vs. ceftiofur (*p* = 0.0018), florfenicol (*p* = 0.0367), tildipirosin (*p* < 0.0001), tilmicosin (*p* = 0.0098). Twelve hours: danofloxacin vs. tildipirosin (*p* = 0.0004); pradofloxacin vs. ceftiofur (*p* = 0.0004), enrofloxacin (*p* = 0.0038), florfenicol (*p* = 0.0048), tildipirosin (*p* < 0.0001), tilmicosin (*p* = 0.0174). Twenty-four hours: danofloxacin vs. florfenicol (*p* = 0.0026), marbofloxacin (*p* < 0.0001), tildipirosin (*p* < 0.0001), ceftiofur (*p* = 0.0019); pradofloxacin vs. ceftiofur (*p* = 0.0001), enrofloxacin (*p* = 0.0058), florfenicol (*p* = 0.0017), marbofloxacin (*p* < 0.0001), tildipirosin (*p* < 0.0001), tilmicosin (*p* = 0.0034), tulathromycin (*p* < 0.0001). (**C**) MPC 10^8^ cfu/mL. Two hours: pradofloxacin vs. ceftiofur (*p* = 0.0222), florfenicol (*p* = 0.0232), marbofloxacin (*p* = 0.0415), tildipirosin (*p* = 0.0040), tulathromycin (*p* = 0.0016). Four hours: pradofloxacin vs. ceftiofur (*p* = 0.0005), enrofloxacin (*p* = 0.0152), florfenicol (*p* = 0.0010), marbofloxacin (*p* < 0.0001), tildipirosin (*p* < 0.0001), tilmicosin (*p* = 0.0023), tulathromycin (*p* = 0.0009). Six hours: pradofloxacin vs. ceftiofur (*p* = 0.0003), danofloxacin (*p* = 0.0051), enrofloxacin (*p* < 0.0001), florfenicol (*p* < 0.0001), marbofloxacin (*p* < 0.0001), tildipirosin (*p* < 0.0001), tilmicosin (*p* = 0.0084), tulathromycin (*p* < 0.0001). Twelve hours: pradofloxacin vs. ceftiofur (*p* = 0.0053), enrofloxacin (*p* = 0.0002), florfenicol (*p* < 0.0001), marbofloxacin (*p* = 0.0003), tildipirosin (*p* < 0.0001), tulathromycin (*p* < 0.0001). Twenty-four hours: pradofloxacin vs. ceftiofur (*p* = 0.0005), danofloxacin (*p* = 0.0002), danofloxacin (*p* = 0.0002), enrofloxacin (*p* < 0.0001), florfenicol (*p* < 0.0001), marbofloxacin (*p* < 0.0001), tildipirosin (*p* < 0.0001), tilmicosin (*p* = 0.0002), tulathromycin (*p* < 0.0001). (**D**) MPC 10^9^ cfu/mL. Twenty-four hours: pradofloxacin vs. enrofloxacin (*p* = 0.0221), florfenicol (*p* < 0.0001), tildipirosin (*p* = 0.0104), tilmicosin (*p* = 0.0309); danofloxacin vs. florfenicol (*p* = 0.0024).

**Figure 3:**
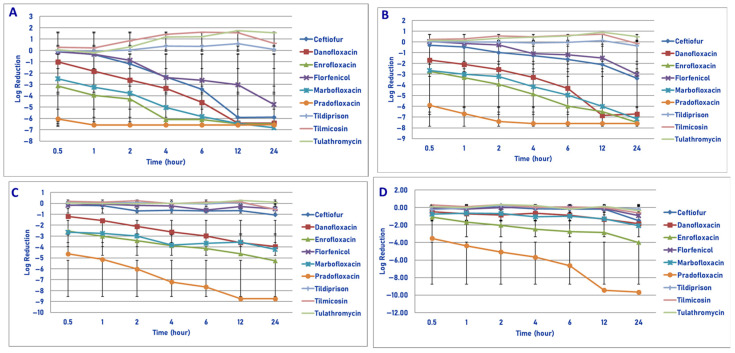
Comparative killing of *Mannheimia haemolytica* by 9 antimicrobial agents at the C_max_: (**A**) 10^6^ cfu/mL, (**B**) 10^7^ cfu/mL, (**C**) 10^8^ cfu/mL, (**D**) 10^9^ cfu/mL drug concentrations. (**A**) C_max_ 10^6^ cfu/mL, 0.5 h: enrofloxacin vs. ceftiofur (*p* = 0.0004), florfenicol (*p* = 0.0004), tildipirosin (*p* = 0.0090), tilmicosin (*p* < 0.0001), tulathromycin (*p* = 0.0002); pradofloxacin vs. ceftiofur (*p* < 0.0001), danofloxacin (*p* < 0.0001), enrofloxacin (*p* = 0.0054), florfenicol (*p* < 0.0001), marbofloxacin (*p* < 0.0001), tildipirosin (*p* < 0.0001), tilmicosin (*p* < 0.0001), tulathromycin (*p* < 0.0001). One hour: enrofloxacin vs. ceftiofur (*p* < 0.0001), florfenicol (*p* < 0.0001), tildipirosin (*p* < 0.0001), tilmicosin (*p* < 0.0001), tulathromycin (*p* < 0.0001); pradofloxacin vs. ceftiofur *(p* < 0.0001), danofloxacin (*p* < 0.0001), enrofloxacin (*p* = 0.0257), florfenicol (*p* < 0.0001), marbofloxacin (*p* < 0.0001), tildipirosin (*p* < 0.0001), tilmicosin (*p* < 0.0001), tulathromycin (*p* < 0.0001); marbofloxacin vs. tildipirosin (*p* = 0.0013), tilmicosin (*p* = 0.0005), tulathromycin (*p* = 0.0068). Two hours: danofloxacin vs. tildipirosin (*p* = 0.0230), tilmicosin (*p* < 0.0001), tulathromycin (*p* < 0.0001); enrofloxacin vs. ceftiofur (*p* = 0.0003), florfenicol (*p* < 0.0001), tildipirosin (*p* < 0.0001), tilmicosin (*p* < 0.0001), tulathromycin (*p* < 0.0001); pradofloxacin vs. ceftiofur (*p* < 0.0001), danofloxacin (*p* < 0.0001), florfenicol (*p* < 0.0001), marbofloxacin (*p* = 0.0019), tildipirosin (*p* < 0.0001), tilmicosin (*p* < 0.0001), tulathromycin (*p* < 0.0001); marbofloxacin vs. florfenicol (*p* = 0.0049), tildipirosin (*p* < 0.0001), tilmicosin (*p* < 0.0001), tulathromycin (*p* < 0.0001). Four hours: danofloxacin vs. tildipirosin (*p* < 0.0002), tilmicosin (*p* < 0.0001), tulathromycin (*p* < 0.0001); enrofloxacin vs. ceftiofur (*p* < 0.0001), danofloxacin (*p* = 0.0056), florfenicol *p* < 0.0001), tildipirosin (*p* < 0.0001), tilmicosin (*p* < 0.0001), tulathromycin (*p* < 0.0001); pradofloxacin vs. ceftiofur (*p* < 0.0001), danofloxacin (*p* = 0.0002), florfenicol (*p* < 0.0001), tildipirosin (*p* < 0.0001), tilmicosin (*p* < 0.0001), tulathromycin (*p* < 0.0001); florfenicol vs. tildipirosin (*p* = 0.0078), tilmicosin (*p* < 0.0001), tulathromycin (*p* < 0.0002); marbofloxacin vs. florfenicol (*p* = 0.0251), tildipirosin (*p* = 0.0078), tilmicosin (*p* < 0.0001), tulathromycin (*p* < 0.0001); ceftiofur vs. tildipirosin (*p* = 0.0125), tilmicosin (*p* < 0.0001), tulathromycin (*p* < 0.0001). Six hours: danofloxacin vs. tildipirosin (*p* < 0.0001), tilmicosin (*p* < 0.0001), tulathromycin (*p* < 0.0001); enrofloxacin vs. ceftiofur (*p* = 0.0073), florfenicol (*p* < −0.0001), tildipirosin (*p* < 0.0001), tilmicosin (*p* < 0.0001), tulathromycin (*p* < 0.0001); florfenicol vs. tildipirosin (*p* = 0.0020), tilmicosin *p* < 0.0001), tulathromycin (*p* < 0.0001); marbofloxacin vs. florfenicol (*p* = 0.0006), tildipirosin (*p* < 0.0001), tilmicosin (*p* < 0.0001), tulathromycin (*p* < 0.0001); pradofloxacin vs. ceftiofur (*p* = 0.0003), florfenicol (*p* < 0.0001), tildipirosin (*p* < 0.0001), tilmicosin (*p* < 0.0001), tulathromycin (*p* < 0.0001); ceftiofur vs. tildipirosin (*p* < 0.0001), tilmicosin (*p* < 0.0001), tulathromycin (*p* < 0.0001). Twelve hours: ceftiofur vs. florfenicol (*p* = 0.0020), tildipirosin (*p* < 0.0001), tilmicosin (*p* < 0.0001), tulathromycin (*p* < 0.0001); danofloxacin vs. florfenicol (*p* < 0.0001), tildipirosin (*p* < 0.0001), tilmicosin (*p* < 0.0001), tulathromycin (*p* < 0. 0001); enrofloxacin vs. florfenicol (*p* < 0.0001), tildipirosin (*p* < 0.0001), tilmicosin (*p* < 0.0001), tulathromycin (*p* < 0.0001); florfenicol vs. tildipirosin (*p* < 0.0001), tilmicosin (*p* < 0.0001), tulathromycin (*p* < 0.0001); marbofloxacin vs. tildipirosin (*p* < 0.0001), tilmicosin (*p* < 0.0001), tulathromycin (*p* < 0.0001); pradofloxacin vs. florfenicol (*p* < 0.0001), tildipirosin (*p* < 0.0001), tilmicosin (*p* < 0.0001), tulathromycin (*p* < 0.0001). Twenty-four hours: ceftiofur, danofloxacin, enrofloxacin, florfenicol, marbofloxacin and pradofloxacin vs. tildipirosin, tilmicosin and tulathromycin (*p* < 0.0001) for all comparisons between the first 6 drugs and the last 3 drugs. (**B**) C_max_ 10^7^ cfu/mL, 0.5 h: pradofloxacin vs. ceftiofur (*p* < 0.0001), danofloxacin (*p* < 0.0001), florfenicol (*p* < 0.0001), enrofloxacin (*p* = 0.0031), marbofloxacin (*p* = 0.0031), tildipirosin (*p* < 0.0001), tilmicosin (*p* < 0.0001), tulathromycin (*p* < 0.0001); enrofloxacin vs. florfenicol (*p* = 0.0310), tilmicosin (*p* = 0.0081), tulathromycin (*p* = 0.0133); marbofloxacin vs. florfenicol (*p* = 0.0474), tilmicosin (*p* = 0.0168), tulathromycin (*p* = 0.0296). One hour: enrofloxacin vs. ceftiofur (*p* = 0.0181), florfenicol (*p* = 0.0025), tilmicosin (*p* < 0.0001), tulathromycin (*p* = 0.0003); marbofloxacin vs. florfenicol (*p* = 0.0155), tilmicosin (*p* = 0.0009), tulathromycin (*p* = 0.0028); pradofloxacin vs. ceftiofur (*p* < 0.0001), danofloxacin (*p* < 0.0001), enrofloxacin (*p* = 0.0009), florfenicol (*p* < 0.0001), marbofloxacin (*p* < 0.0001), tildipirosin (*p* < 0.0001), tilmicosin (*p* < 0.0001), tulathromycin (*p* < 0.0001). Two hours: danofloxacin vs. tilmicosin (*p* = 0.0371); enrofloxacin vs. ceftiofur (*p* = 0.0081), florfenicol (*p* < 0.0001), tildipirosin (*p* = 0.0119), tilmicosin (*p* < 0.0001), tulathromycin (*p* < 0.0001); marbofloxacin vs. tilmicosin (*p* < 0.0001), tulathromycin (*p* = 0.0002); pradofloxacin vs. ceftiofur (*p* < 0.0001), danofloxacin (*p* < 0.0001), enrofloxacin (*p* = 0.0004), florfenicol (*p* < 0.0001), tildipirosin (*p* < 0.0001), tilmicosin (*p* < 0.0001), tulathromycin (*p* < 0.0001). Four hours: danofloxacin vs. tilmicosin (*p* = 0.0006), tulathromycin (*p* = 0.0008); enrofloxacin vs. ceftiofur (*p* = 0.0001), florfenicol (*p* < 0.0001), tildipirosin (*p* < 0.0001), tilmicosin (*p* < 0.0001), tulathromycin (*p* < 0.0001); marbofloxacin vs. ceftiofur (*p* = 0.0134), florfenicol (*p* < 0.0041), tildipirosin (*p* < 0.0001), tilmicosin (*p* < 0.0001), tulathromycin (*p* < 0.0001); pradofloxacin vs. ceftiofur (*p* < 0.0001), danofloxacin (*p* < 0.0001), enrofloxacin (*p* = 0.0243), florfenicol (*p* < 0.0001), marbofloxacin (*p* = 0.0002), tildipirosin (*p* < 0.0001), tilmicosin (*p* < 0.0001), tulathromycin (*p* < 0.0001). Six hours: danofloxacin vs. tildipirosin (*p* = 0.0112), tilmicosin (*p* < 0.0001), tulathromycin (*p* < 0.0001); enrofloxacin vs. ceftiofur (*p* < 0.0001), florfenicol (*p* < 0.0001), tildipirosin (*p* < 0.0001), tilmicosin (*p* < 0.0001), tulathromycin (*p* < 0.0001); marbofloxacin vs. ceftiofur (*p* = 0.0006), tildipirosin (*p* < 0.0001), tilmicosin (*p* < 0.0001), tulathromycin (*p* < 0.0001); pradofloxacin vs. ceftiofur (*p* < 0.0001), danofloxacin (*p* = 0.0075), florfenicol (*p* < 0.0001), marbofloxacin (*p* = 0.0371), tildipirosin (*p* < 0.0001), tilmicosin (*p* < 0.0001), tulathromycin (*p* < 0.0001). Twelve hours: danofloxacin vs. ceftiofur (*p* < 0.0001), florfenicol (*p* < 0.0001), tildipirosin (*p* < 0.0001), tilmicosin (*p* < 0.0001), tulathromycin (*p* < 0.0001); ceftiofur vs. tilmicosin (*p* = 0.0199), tulathromycin (*p* = 0.061); enrofloxacin vs. ceftiofur (*p* < 0.0001), florfenicol (*p* < 0.0001), tildipirosin (*p* < 0.0001), tilmicosin (*p* < 0.0001), tulathromycin (*p* < 0.0001); marbofloxacin vs. florfenicol (*p* < 0.0001), tildipirosin (*p* < 0.0001), tilmicosin (*p* < 0.0001), tulathromycin (*p* < 0.0001); pradofloxacin vs. ceftiofur (*p* < 0.0001), florfenicol (*p* < 0.0001), tildipirosin (*p* < 0.0001), tilmicosin (*p* < 0.0001), tulathromycin (*p* < 0.0001). Twenty-four hours: danofloxacin vs. ceftiofur (*p* = 0.0170), florfenicol (*p* = 0.0022), tildipirosin (*p* < 0.0001), tilmicosin (*p* < 0.0001), tulathromycin (pp < 0.0001); ceftiofur vs. tilmicosin (*p* = 0.0011), tulathromycin (*p* < 0.0001); enrofloxacin vs. ceftiofur (*p* < 0.0001), florfenicol (*p* < 0.0001), tildipirosin (*p* < 0.0001), tilmicosin (*p* < 0.0001), tulathromycin (*p* < 0.0001); florfenicol vs. tilmicosin (*p* = 0.0154), tulathromycin (*p* = 0.0002); marbofloxacin vs. ceftiofur (*p* < 0.0001), florfenicol (*p* < 0.0001), tildipirosin (*p* < 0.0001), tilmicosin (*p* < 0.0001), tulathromycin (*p* < 0.0001); pradofloxacin vs. ceftiofur (*p* < 0.0001), florfenicol (*p* < 0.0001), tildipirosin (*p* < 0.0001), tilmicosin (*p* < 0.0001), tulathromycin (*p* < 0.0001). (**C**) C_max_ 10^8^ cfu/mL, 0.5 h: enrofloxacin vs. tilmicosin (*p* = 0.0179); marbofloxacin vs. tildipirosin (*p* = 0.0179), tilmicosin (*p* = 0.0172); pradofloxacin vs. ceftiofur (*p* < 0.0001), danofloxacin (*p* = 0.0016), florfenicol (*p* < 0.0001), tildipirosin (*p* < 0.0001), tilmicosin (*p* < 0.0001), tulathromycin (*p* < 0.0001). One hour: enrofloxacin vs. ceftiofur (*p* = 0.0122), florfenicol (*p* = 0.0067), tildipirosin (*p* = 0.0059), tilmicosin (*p* = 0.0013), tulathromycin (*p* = 0.0348); marbofloxacin vs. tildipirosin (*p* = 0.0123), tilmicosin *p* = 0.0174); pradofloxacin vs. ceftiofur (*p* < 0.0001), danofloxacin (*p* = 0.0007), florfenicol (*p* < 0.0001), tildipirosin (*p* < 0.0001), tilmicosin (*p* < 0.0001), tulathromycin (*p* < 0.0001). Two hours: enrofloxacin vs. ceftiofur (*p* = 0.0020), florfenicol (*p* = 0.0009), tildipirosin (*p* = 0.0003), tilmicosin (*p* < 0.0001), tulathromycin (*p* = 0.0013); marbofloxacin vs. florfenicol (*p* = 0.0099), tildipirosin (*p* = 0.0017), tilmicosin (*p* = 0.0012), tulathromycin (*p* = 0.0136); pradofloxacin vs. ceftiofur (*p* < 0.0001), danofloxacin (*p* < 0.0001), enrofloxacin (*p* = 0.0455), florfenicol (*p* < 0.0001), marbofloxacin (*p* = 0.0029), tildipirosin (*p* < 0.0001), tilmicosin (*p* < 0.0001), tulathromycin (*p* < 0.0001). Four hours: enrofloxacin vs. ceftiofur (*p* < 0.0001), florfenicol (*p* < 0.0001), tildipirosin (*p* < 0.0001), tilmicosin (*p* < 0.0001), tulathromycin (*p* = 0.0003); marbofloxacin vs. ceftiofur (*p* = 0.0003), florfenicol (*p* < 0.0001), tildipirosin (*p* < 0.0001), tilmicosin (*p* < 0.0001), tulathromycin (*p* = 0.0003); pradofloxacin vs. ceftiofur (*p* < 0.0001), danofloxacin (*p* < 0.0001), enrofloxacin (*p* = 0.0003), florfenicol (*p* < 0.0001), marbofloxacin (*p* = 0.0002), tildipirosin (*p* < 0.0001), tilmicosin (*p* < 0.0001), tulathromycin (*p* < 0.0001). Six hours: danofloxacin vs. tilmicosin (*p* = 0.0080); enrofloxacin vs. ceftiofur (*p* < 0.0001), florfenicol (*p* < 0.0001), tildipirosin (*p* < 0.0001), tilmicosin (*p* < 0.0001), tulathromycin (*p* < 0.0001); marbofloxacin vs. ceftiofur (*p* = 0.0010), florfenicol (*p* = 0.0021), tildipirosin (*p* < 0.0001), tilmicosin (*p* < 0.0001), tulathromycin (*p* = 0.0006); pradofloxacin vs. ceftiofur (*p* < 0.0001), danofloxacin (*p* < 0.0001), enrofloxacin (*p* = 0.0002), florfenicol (*p* < 0.0001), marbofloxacin (*p* < 0.0001), tildipirosin (*p* < 0.0001), tilmicosin (*p* < 0.0001), tulathromycin (*p* < 0.0001). Twelve hours: danofloxacin vs. ceftiofur (*p* = 0.0020), florfenicol *p* = 0.0097), tildipirosin (*p* = 0.0025), tilmicosin (*p* = 0.0001), tulathromycin (*p* = 0.0027); enrofloxacin vs. ceftiofur (*p* < 0.0001), florfenicol (*p* < 0.0001), tildipirosin (*p* < 0.0001), tilmicosin (*p* < 0.0001), tulathromycin (*p* < 0.0001); marbofloxacin vs. ceftiofur (*p* = 0.0010), florfenicol (*p* = 0.0021), tildipirosin (*p* < 0.0001), tilmicosin (*p* < 0.0001), tulathromycin (*p* = 0.0006); pradofloxacin vs. ceftiofur (*p* < 0.0001), danofloxacin (*p* < 0.0001), enrofloxacin (*p* = 0.0002), florfenicol (*p* < 0.0001), marbofloxacin (*p* < 0.0001), tildipirosin (*p* < 0.0001), tilmicosin (*p* < 0.0001), tulathromycin (*p* < 0.0001). Twenty-four hours: danofloxacin vs. ceftiofur (*p* = 0.0025), florfenicol (*p* = 0.0046), tildipirosin (*p* = 0.0039), tilmicosin (*p* = 0.0012), tulathromycin (*p* = 0.0013); enrofloxacin vs. ceftiofur (*p* < 0.0001), florfenicol (*p* < 0.0001), tildipirosin (*p* < 0.0001), tilmicosin (*p* < 0.0001); marbofloxacin vs. ceftiofur (*p* = 0.0004), florfenicol (*p* < 0.0001), tildipirosin (*p* < 0.0001), tilmicosin (*p* < 0.0001), tulathromycin (*p* < 0.0001); pradofloxacin vs. ceftiofur (*p* < 0.0001), danofloxacin (*p* < 0.0001), enrofloxacin (*p* < 0.0001), florfenicol (*p* < 0.0001), marbofloxacin (*p* < 0.0001), tildipirosin (*p* < 0.0001), tilmicosin (*p* < 0.0001), tulathromycin (*p* < 0.0001). (**D**) C_max_ 10^9^ cfu/mL, 0.5 h: pradofloxacin vs. ceftiofur (*p* = 0.0001), danofloxacin (*p* = 0.0017), enrofloxacin (*p* = 0.0173), florfenicol (*p* < 0.0001), marbofloxacin (*p* = 0.0011), tildipirosin (*p* < 0.0001), tilmicosin (*p* < 0.0001), tulathromycin (*p* < 0.0001). One hour: pradofloxacin vs. ceftiofur (*p* < 0.0001), danofloxacin (*p* < 0.0001), enrofloxacin (*p* = 0.0025), florfenicol (*p* < 0.0001), marbofloxacin (*p* < 0.0001), tildipirosin (*p* < 0.0001), tilmicosin (*p* < 0.0001), tulathromycin (*p* < 0.0001). Two hour: pradofloxacin vs. ceftiofur (*p* < 0.0001), danofloxacin (*p* < 0.0001), enrofloxacin (*p* = 0.0020), florfenicol (*p* < 0.0001), marbofloxacin (*p* < 0.0001), tildipirosin (*p* < 0.0001), tilmicosin (*p* < 0.0001), tulathromycin (*p* < 0.0001); enrofloxacin vs. tilmicosin (*p* = 0.0210). Four h: enrofloxacin vs. tilmicosin (*p* = 0.0083); pradofloxacin vs. ceftiofur (*p* < 0.0001), danofloxacin (*p* < 0.0001), enrofloxacin (*p* < 0.0001), florfenicol (*p* < 0.0001), marbofloxacin (*p* < 0.0001), tildipirosin (*p* < 0.0001), tilmicosin (*p* < 0.0001), tulathromycin (*p* < 0.0001). Six hours: enrofloxacin vs. ceftiofur (*p* = 0.0404), florfenicol (*p* = 0.0014), tildipirosin (*p* = 0.0285), tilmicosin (*p* = 0.0009); pradofloxacin vs. ceftiofur (*p* < 0.0001), danofloxacin (*p* < 0.0001), enrofloxacin (*p* < 0.0001), florfenicol (*p* < 0.0001), marbofloxacin (*p* < 0.0001), tildipirosin (*p* < 0.0001), tilmicosin (*p* < 0.0001), tulathromycin (*p* < 0.0001). Twelve hours: enrofloxacin vs. ceftiofur (*p* = 0.0245), florfenicol *p* = 0.0020), tildipirosin (*p* = 0.0080), tilmicosin (*p* = 0.0006), tulathromycin (*p* = 0.0185); pradofloxacin vs. ceftiofur (*p* < 0.0001), danofloxacin (*p* < 0.0001), enrofloxacin (*p* < 0.0001), florfenicol (*p* < 0.0001), marbofloxacin (*p* < 0.0001), tildipirosin (*p* < 0.0001), tilmicosin (*p* < 0.0001), tulathromycin (*p* < 0.0001). Twenty-four hours: enrofloxacin vs. florfenicol (*p* = 0.0002), tildipirosin (*p* < 0.0001), tilmicosin (*p* < 0.0001), tulathromycin (*p* = 0.0009); pradofloxacin vs. ceftiofur (*p* < 0.0001), danofloxacin (*p* < 0.0001), enrofloxacin (*p* < 0.0001), florfenicol (*p* < 0.0001), marbofloxacin (*p* < 0.0001), tildipirosin (*p* < 0.0001), tilmicosin (*p* < 0.0001), tulathromycin (*p* < 0.0001).

**Figure 4:**
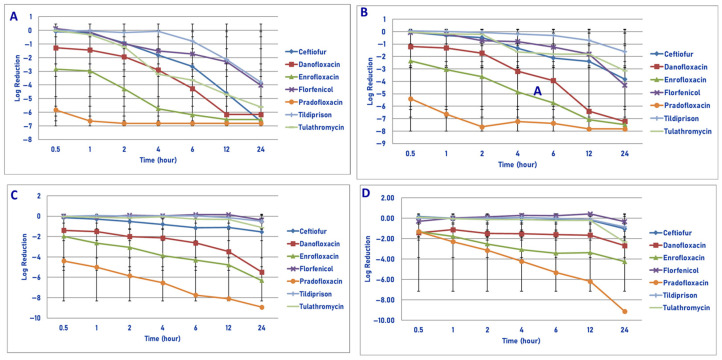
Comparative killing of *Mannheimia haemolytica* by 7 antimicrobial agents at the T_issuemax_: (**A**) 10^6^ cfu/mL, (**B**) 10^7^cfu/mL, (**C**) 10^8^ cfu/mL, (**D**) 10^9^ cfu/mL drug concentrations. (**A**) T_issuemax_ 10^6^ cfu/mL, 0.5 h: enrofloxacin vs. ceftiofur (*p* = 0.0052), florfenicol (*p* = 0.0061), tildipirosin (*p* = 0.0003), tulathromycin (*p* = 0.0022); pradofloxacin vs. ceftiofur (*p* < 0.0001), danofloxacin (*p* = 0.0003), florfenicol (*p* < 0.0001), tildipirosin (*p* < 0.0001), tulathromycin (*p* < 0.0001). One hour: enrofloxacin vs. ceftiofur (*p* = 0.0054), florfenicol (*p* = 0.0169), tildipirosin (*p* < 0.0001), tulathromycin (*p* = 0.0069); pradofloxacin vs. ceftiofur (*p* < 0.0001), danofloxacin (*p* < 0.0001), florfenicol (*p* < 0.0001), tildipirosin (*p* < 0.0001), tulathromycin (*p* < 0.0001). Two hours: enrofloxacin vs. ceftiofur (*p* < 0.0001), florfenicol (*p* = 0.0008), tildipirosin (*p* < 0.0001), tulathromycin (*p* < 0.0001); pradofloxacin vs. ceftiofur (*p* < −0.0001), danofloxacin (*p* < 0.0001), florfenicol (*p* < 0.0001), tildipirosin (*p* < 0.0001), tulathromycin (*p* < 0.0001). Four hours: danofloxacin vs. tildipirosin (*p* = 0.0006); enrofloxacin vs. ceftiofur (*p* < 0.0001), florfenicol (*p* < 0.0001), tildipirosin (*p* < 0.0001), tulathromycin (*p* = 0.0154); pradofloxacin vs. ceftiofur (*p* < 0.0001), danofloxacin (*p* = 0.0114), florfenicol (*p* < 0.0001), tildipirosin (*p* < 0.0001), tulathromycin (*p* = 0.0003); tulathromycin vs. tildipirosin (*p* = 0.0021). Six hours: danofloxacin vs. florfenicol (*p* = 0.0142), tildipirosin (*p* < 0.0001); enrofloxacin vs. ceftiofur (*p* < 0.0001), florfenicol (*p* < 0.0001), tildipirosin (*p* < 0.0001), tulathromycin (*p* = 0.0137); pradofloxacin vs. ceftiofur (*p* < 0.0001), florfenicol (*p* < 0.0001), tildipirosin (*p* < 0.0001), tulathromycin (*p* = 0.0056); tulathromycin vs. tildipirosin (*p* = 0.0022). Twelve hours: ceftiofur vs. tildipirosin (*p* = 0.0188); danofloxacin vs. florfenicol (*p* < 0.0001), tildipirosin (*p* < 0.0001); enrofloxacin vs. florfenicol (*p* < 0.0001), tildipirosin (*p* < 0.0001); pradofloxacin vs. florfenicol (*p* < 0.0001), tildipirosin (*p* < 0.0001); tulathromycin vs. tildipirosin (*p* = 0.0162). Twenty-four hours: ceftiofur vs. tildipirosin (*p* = 0.0016); danofloxacin vs. tildipirosin (*p* = 0.0026); enrofloxacin vs. tildipirosin (*p* = 0.0015); pradofloxacin vs. tildipirosin (*p* = 0.0048). (**B**) T_issuemax_ 10^7^ cfu/mL, 0.5 h: pradofloxacin vs. ceftiofur (*p* < 0.0001), danofloxacin (*p* = 0.0309), florfenicol (*p* < 0.0001), tildipirosin (*p* < 0.0001), tulathromycin (*p* < 0.0001). One hour: enrofloxacin vs. tildipirosin (*p* = 0.0198); pradofloxacin vs. ceftiofur (*p* < 0.0001), danofloxacin (*p* = 0.0002), florfenicol (*p* < 0.0001), tildipirosin (*p* < 0.0001), tulathromycin (*p* < 0.0001). Two hours: enrofloxacin vs. ceftiofur (*p* = 0.0187), tildipirosin (*p* = 0.0017), tulathromycin (*p* = 0.0047); pradofloxacin vs. ceftiofur (*p* < 0.0001), danofloxacin (*p* < 0.0001), florfenicol (*p* < 0.0001), tildipirosin (*p* < 0.0001), tulathromycin (*p* < 0.0001). Four hours: enrofloxacin vs. ceftiofur (*p* = 0.0029), florfenicol (*p* = 0.0004), tildipirosin (*p* < 0.0001), tulathromycin (*p* = 0.0109); pradofloxacin vs. ceftiofur (*p* < 0.0001), danofloxacin (*p* = 0.0030), florfenicol (0 < 0.0001), tildipirosin (*p* < 0.0001), tulathromycin (*p* < 0.0001). Six hours: danofloxacin vs. tildipirosin (*p* = 0.0097); enrofloxacin vs. ceftiofur (*p* < 0.0001), florfenicol (*p* < 0.0001), tildipirosin (*p* < 0.0001), tulathromycin (*p* = 0.0003); pradofloxacin vs. ceftiofur (*p* < 0.0001), florfenicol (*p* < 0.0001), tildipirosin (*p* < 0.0001), tulathromycin (*p* < 0.0001). Twelve hours: danofloxacin vs. ceftiofur (*p* = 0.0010), florfenicol (*p* < 0.0001), tildipirosin (*p* < 0.0001), tulathromycin (*p* < 0.0001); enrofloxacin vs. ceftiofur (*p* < 0.0001), florfenicol (*p* < 0.0001), tildipirosin (*p* < 0.0001), tulathromycin (*p* < 0.0001); pradofloxacin vs. ceftiofur (*p* < 0.0001), florfenicol (*p* < 0.0001), tildipirosin (*p* < 0.0001), tulathromycin (*p* < 0.0001). Twenty-four hours: danofloxacin vs. ceftiofur (*p* = 0.0175), tildipirosin (*p* < 0.0001), tulathromycin (*p* = 0.0007); enrofloxacin vs. ceftiofur (*p* = 0.0014), florfenicol (*p* = 0.0367), tildipirosin (*p* < 0.0001), tulathromycin (*p* < 0.0001); pradofloxacin vs. ceftiofur (*p* = 0.0016), tildipirosin (*p* < 0.0001), tulathromycin (*p* < 0.0001). (**C**) T_issuemax_ 10^8^ cfu/mL, 0.5 h: pradofloxacin vs. ceftiofur (*p* < 0.0001), florfenicol (*p* < 0.0001), tildipirosin (*p* < 0.0001), tulathromycin (*p* < 0.0001). One hour: danofloxacin vs. ceftiofur (*p* = 0.0034), florfenicol (*p* = 0.0026), tildipirosin (*p* = 0.0004), tulathromycin (*p* = 0.0016); enrofloxacin vs. ceftiofur (*p* = 0.0176), florfenicol (*p* = 0.0132), tildipirosin (*p* = 0.0010), tulathromycin (*p* = 0.0068); pradofloxacin vs. ceftiofur (*p* < 0.0001), florfenicol (*p* < 0.0001), tildipirosin (*p* < 0.0001), tulathromycin (*p* < 0.0001). Two hours: enrofloxacin vs. ceftiofur (*p* = 0.0075), florfenicol (*p* = 0.0003), tildipirosin (*p* = 0.0002), tulathromycin (*p* = 0.0008); pradofloxacin vs. ceftiofur (*p* < 0.0001), florfenicol (*p* < 0.0001), tildipirosin (*p* < 0.0001), tulathromycin (*p* < 0.0001). Four hours: enrofloxacin vs. ceftiofur (*p* = 0.0001), florfenicol (*p* < 0.0001), tildipirosin (*p* < 0.0001), tulathromycin (*p* < 0.0001); pradofloxacin vs. ceftiofur (*p* < 0.0001), danofloxacin (*p* = 0.0137), florfenicol (*p* < 0.0001), tildipirosin (*p* < 0.0001), tulathromycin (*p* < 0.0001). Six hours: enrofloxacin vs. ceftiofur (*p* < 0.0001), danofloxacin (*p* = 0.0002), florfenicol (*p* < 0.0001), tildipirosin (*p* < 0.0001), tulathromycin (*p* < 0.0001); pradofloxacin vs. ceftiofur (*p* < 0.0001), florfenicol (*p* < 0.0001), tildipirosin (*p* < 0.0001), tulathromycin (*p* < 0.0001). Twelve hours: enrofloxacin vs. ceftiofur (*p* < 0.0001), florfenicol (*p* < 0.0001), tildipirosin (*p* < 0.0001), tulathromycin (*p* < 0.0001); pradofloxacin vs. ceftiofur (*p* < 0.0001), danofloxacin (*p* = 0.0021), tulathromycin (*p* < 0.0001). Twenty-four hours: danofloxacin vs. florfenicol (*p* = 0.0037), tildipirosin (*p* = 0.0054), tulathromycin (*p* = 0.0235); enrofloxacin vs. ceftiofur (*p* < 0.0001), florfenicol (*p* < 0.0001), tildipirosin (*p* < 0.0001), tulathromycin (*p* < 0.0001); pradofloxacin vs. ceftiofur (*p* < 0.0001), tulathromycin (*p* < 0.0001). (**D**) T_issuemax_ 10^9^ cfu/mL. One hour: pradofloxacin vs. tildipirosin (*p* = 0.0413). Two hours: enrofloxacin vs. ceftiofur (*p* = 0.0056), florfenicol (*p* = 0.0106), tildipirosin (*p* = 0.0014), tulathromycin (*p* = 0.0112); pradofloxacin vs. ceftiofur (*p* = 0.0013), florfenicol (*p* = 0.0086), tildipirosin (*p* < 0.0001), tulathromycin (*p* = 0.0030). Four hours: enrofloxacin vs. ceftiofur (*p* = 0.0002), florfenicol (*p* < 0.0001), tildipirosin (*p* < 0.0001), tulathromycin (*p* = 0.0002); pradofloxacin vs. ceftiofur (*p* < 0.0001), florfenicol (*p* < 0.0001), tildipirosin (*p* < 0.0001), tulathromycin (*p* < 0.0001). Six hours: enrofloxacin vs. ceftiofur (*p* < 0.0001), florfenicol (*p* < 0.0001), tildipirosin (*p* < 0.0001), tulathromycin (*p* < 0.0001); pradofloxacin vs. ceftiofur (*p* < 0.0001), danofloxacin (*p* = 0.0006), florfenicol (*p* < 0.0001), tildipirosin (*p* < 0.0001), tulathromycin (*p* < 0.0001). Twelve hours: enrofloxacin vs. ceftiofur (*p* = 0.0004), florfenicol (*p* < 0.0001), tildipirosin (*p* = 0.0001), tulathromycin (*p* = 0.0005); pradofloxacin vs. ceftiofur (*p* < 0.0001), danofloxacin (*p* = 0.0006), florfenicol (*p* < 0.0001), tildipirosin (*p* < 0.0001), tulathromycin (*p* < 0.0001). Twenty-four hours: enrofloxacin vs. ceftiofur (*p* < 0.0001), florfenicol (*p* < 0.0001), tildipirosin (*p* < 0.0001); pradofloxacin vs. ceftiofur (*p* < 0.0001), danofloxacin (*p* < 0.0001), florfenicol (*p* < 0.0001), tildipirosin (*p* < 0.0001), tulathromycin (*p* < 0.0001).

Exposure of 10^6^ cfu/mL of *M. haemolytica* to MIC drug concentrations for the eight antimicrobial agents did not result in any statistical differences in kill between the agents following 30 min, 1 h and 2 h of drug exposure (Figure 1A). Pradofloxacin killed 61.9% of cells following 1 h of drug exposure, 92% following 2 h and 99.99% following 12 h. By comparison, the killing by ceftiofur over these same time frames was <1%, 15.1% and 99.5%, respectively. Danofloxacin and enrofloxacin killed 48.2% and 65.9% of cells, respectively, following 24 h of drug exposure. For the remaining drugs, killing up to 12.8% occurred after 1 h of drug exposure but then growth (organism growth in the presence of the drug) thereafter. Statistically significant differences in killing between drugs and at the various drug concentrations tested are highlighted below eachfigure (Figure 1, Figure 2, Figure 3 and Figure 4).

Exposure of 10^7^ cfu/mL of *M. haemolytica* to the MIC drug concentration of the eight drugs tested showed no significant difference in killing between the drugs following 30 min, 1, 2 and 4 h of drug exposure (Figure 1B). Pradofloxacin killed 64.4% of cells following 2 h of drug exposure, which increased to a 93.7–95.0% kill following 4–6 h of drug exposure and a 98.4–98.8% kill following 12–24 h of drug exposure. Ceftiofur killed 51.9–73.9% of cells following 4–6 h of drug exposure and 91.2–96.1% of cells following 12–24 h of drug exposure. Danofloxacin killed 36.3% of cells following 24 h of drug exposure. Killing in the presence of the other drugs was minimal (7–23%), with growth at most time points.

Exposure of 10^8^ cfu/mL of *M. haemolytica* to the eight antimicrobial agents tested did not result in any significant differences in killing between the agents following 30 min, 1, 2 and 6 h of drug exposure (Figure 1C). Pradofloxacin killed 43.5–67.4% of cells following 1–6 h of drug exposure and 94.1–95.4% of cells following 12–24 h of drug exposure. Ceftiofur, enrofloxacin, marbofloxacin and tilmicosin killed 44.8–69.9% of cells following 24 h of drug exposure. Danofloxacin killed 27.38% of cells following 12 h of drug exposure. Growth occurred in the presence of florfenicol at all time points. Tulathromycin killed 15.1% of cells following 24 h of drug exposure.

Following exposure of 10^9^ cfu/mL of *M. haemolytica* to the eight antimicrobial drugs tested, statistical differences in kill between the drugs were not seen after 0.5, 1, 2, 4, 6 and 12 h of drug exposure (Figure 1D). Following 24 h of drug exposure, pradofloxacin (2.3 log_10_, 95.0% kill) killed more cells than did danofloxacin (0.6 log_10_, 69.7% kill, *p* < 0.0001), florfenicol (0.6 log_10_, 74.2% kill, *p* = 0.0385), tildipirosin (0.7 log_10_, 79.8% kill, *p* = 0.0003), tilmicosin (0.7 log_10_, 78.4% kill, *p* = 0.125) and tulathromycin (0.5 log_10_, 58.0% kill, *p* = 0.0133).

In considering time, statistically significant differences were seen for treatment (0.0125–0.003), time (0.001–<0.0001) and treated by time (0.0240–<0.0001) for 10^6^, 10^7^, 10^8^ and 10^9^ cfu/mL exposed to the MIC drug concentration.

#### 3.1.2. Exposure to Mutant Prevention Drug Concentration (MPC)

Exposure of 10^6^ cfu/mL to the MPC drug concentrations of the antibiotics tested did not show any significant differences in kill between agents following 30 min and 1 h of drug exposure (Figure 2A). Pradofloxacin killed 89.4% of cells following 30 min of drug exposure, 98.7–99.99% following 1–4 h of drug exposure and 99.99–100% of cells following 6–12 h of drug exposure. Ceftiofur killed 4.8–56.1% of cells following 30 min–2 h of drug exposure and 92.6–99.9% following 4–12 h of drug exposure; 99.99% following 24 h of drug exposure. Danofloxacin killed 69% of cells following 30 min, 92.8–99.9% following 1–4 h and 100% following 12 h of drug exposure. Enrofloxacin killed 38.5–99.9% of cells following 30 min–4 h of drug exposure and 99.98–99.99% following 12–24 h of drug exposure. Marbofloxacin killed up to 99.8% of cells following 6 h of drug exposure and regrowth occurred between 6–12 and 12–24 h. Tildipirosin killed up to 86.9% of cells following 12 h of drug exposure but regrowth occurred between 12 and 24 h. Tilmicosin and tulathromycin killed up to 97.3–99.9% of cells following 4–6 h of drug exposure, however, regrowth occurred in the presence of tulathromycin from 6 to 12 and from 12 to 24 h.

Exposure of 10^7^ cfu/mL to the MPC drug concentration of the eight drugs tested did not show any significant differences in kill between the drugs following 30 min and 1 h of drug exposure (Figure 2B). Pradofloxacin killed 95.0% of cells following 30 min of drug exposure, 99.6% following 1 h and 99.9–100% at all time points thereafter. Danofloxacin killed 88.1% of cells at 30 min and 79.9% following 4 h of drug exposure. By comparison, ceftiofur, enrofloxacin and marbofloxacin killed 90.6–99.5% of cells following 6 h of drug exposure and 94.4–99.9% following 24 h. Tildipirosin killed 49.6% of cells following 24 h and tilmicosin and tulathromycin killed 96.2–99.2% of cells following 6–12 h of drug exposure and 90.9–99.2% of cells following 6–12 h of drug exposure and 90.9–95.6% following 24 h.

Following exposure of 10^8^ cfu/mL to the antibiotic MPC drug concentration, statistically significant differences in killing between the agents was not seen following 0.5 and 1 h of drug exposure (Figure 2C). Danofloxacin killed 72.8% of cells following 30 min of drug exposure which increased to 92% by 1 h following which 68.4–70.8% of cells were killed following 12–24 h of drug exposure. Pradofloxacin killed 84.3% of cells following 30 min of drug exposure 94.4–99.3% following 1–6 h of drug exposure and 99.9% following 24 h. Ceftiofur killed 92.2% of cells following 24 h as compared to 38.3–65.1% for enrofloxacin and marbofloxacin and 39.8–84.6% for tildipirosin, tilmicosin and tulathromycin.

Exposure of 10^9^ cfu/mL to the MPC drug concentration for the eight antimicrobial agents did not result in significant differences in kill between the drugs following 0.5, 1, 2, 4, 6 and 12 h of drug exposure (Figure 2D). Pradofloxacin killed 63.7% of cells following 12 h of drug exposure and 98.9% following 24 h. Danofloxacin killed 58.9% of cells by 4 h and 80.4–98.2% of cells following 12–24 h of drug exposure. The remaining agents killed between growth −67.0% of cells following 12 h and 66.9–94.9% of cells following 24 h of drug exposure. Following 24 h of drug exposure, pradofloxacin (2.3 log_10_, 98.9% kill) killed more cells than did enrofloxacin (0.9 log_10_, 76.3% kill, *p* = 0.0221), florfenicol (0.5 log_10_, 66.9% kill, *p* < 0.0001), tildipirosin (1.0 log_10_, 76.2% kill, *p* = 0.0104) and tilmicosin (1.4 log_10_, 93.6% kill, *p* = 0.0309). Tulathromycin (1.9 log_10_, 90.1% kill) killed more cells than did florfenicol (*p* = 0.0148).

In considering time, statistically significant differences were seen for treatment (*p* = 0.0156–<0.0001), time (<0.0001 for all comparisons) and treatment by time (0.002–<0.0001) for the 10^6^, 10^7^, 10^8^ and 10^9^ cfu/mL exposed to the MPC drug concentrations.

#### 3.1.3. Exposure to Maximum Serum Total Drug Concentration (C_max_)

Exposure of 10^6^ cfu/mL of *M. haemolytica* to the C_max_ drug concentration of each of the 9 drugs tested yielded the following results (Figure 3A). Pradofloxacin killed 99.99% of cells following 30 min of drug exposure and 100% following 1 h and all time points thereafter. Enrofloxacin killed 78.7% of cells following 30 min, 99.9% following 2 h and 100% following 4 h and all time points thereafter. Danofloxacin killed 98.4% of cells following 1 h and ≥99%–100% of cells following 2–12 h of drug exposure. Marbofloxacin killed 93.7% of cells following 30 min, 99.99% following 4 h and 100% following 12 and 24 h of drug exposure. Ceftiofur killed 16% of cells following 30 min of drug exposure, 99.6% following 6 h and 99.99% and 100% following 12 and 24 h, respectively. For tildipirosin, tilmicosin and tulathromycin, killing rates from growth to 34.2% kill occurred following 30 min and 1 h of drug exposure following which growth occurred in the presence of each drug.

Exposure of 10^7^ cfu/mL to the C_max_ drug concentration of the 9 drugs tested yielded the following results (Figure 3B). Pradofloxacin killed 99.99% of cells following 30 min and 1 h of drug exposure and 100% of cells following 2 h and all time points thereafter. Danofloxacin killed 90.1% of cells at 30 min as compared to 99.9% at 6 h and 100% following 12 and 24 h of drug exposure. Enrofloxacin and marbofloxacin killed 99.4–99.9% of cells following 30 min and 1 h of drug exposure and 99.9–99.99% between 2 and 4 h of drug exposure and 100% following 24 h of drug exposure. Florfenicol killed 59.6% and 87.3% of cells following 4 and 6 h of drug exposure and 97.1% of cells following 24 h of drug exposure. Ceftiofur killed between 48.9–88.9% of cells following 30 min–2 h of drug exposure, 98.8% following 12 h and 99.9% following 24 h. Tildipirosin killed 55.8% of cells following 24 h of drug exposure and growth −19.9% kill occurred for tilmicosin and tulathromycin.

Following exposure of 10^8^ cfu/mL of *M. haemolytica* to the maximum serum drug concentration, pradofloxacin killed 99.99% of cells following 30 min of drug exposure and thereafter until 12 h when 100% of cells were killed (Figure 3C). By comparison, danofloxacin, enrofloxacin and marbofloxacin killed 91.8–99.4% of cells following 30 min of drug exposure which increased to 98.6–99.99% kill following 4–6 h of drug exposure. For ceftiofur, 21.1% of cells were killed following 30 min of drug exposure which increased to 74.8% kill by 4 h and 83.6% kill following 24 h of drug exposure. Killing shown for florfenicol, tildipirosin and tilmicosin where the percentage of cells killed following 24 h of drug exposure ranged from 48.8 to 72.1%. For tulathromycin, the maximum percentage of cells killed was 31.7% following 4 h of drug exposure following which there was regrowth.

Exposure of 10^9^ cfu/mL of *M. haemolytica* to the maximum serum concentration of the drugs tested yielded the following observation (Figure 3D). Pradofloxacin killed 99.9% of cells following 30 min of drug exposure, 99.99% following 1 h and 100% of cells following 12 h of drug exposure. Danofloxacin killed 63.4% of cells at 30 min, 72.7% of cells at 6 h and 83.6 and 89.2% of cells at 12 and 12 h, respectively. Enrofloxacin killed 82.2% of cells and marbofloxacin killed 45.9% of cells following 30 min of drug exposure. Enrofloxacin killed 99.6% of cells following 4 h of drug exposure and 99.9% following 24 h. Marbofloxacin killed 73.2% of cells following 4 h and 94.5% of cells following 24 h of drug exposure. Ceftiofur killed 27.9% of cells following 30 min, 30.3% of cells following 6 h and 96.0% of cells following 24 h of drug exposure. Florfenicol, tildipirosin and tulathromycin killed between 25.8–88.1% of cells following 24 h of drug exposure. Tulathromycin killed 3.6% of cells following 6 h of drug exposure, and growth occurred at all other time points. At all time points, pradofloxacin (3.5–9.7 log_10_, 99.9–100% kill) killed more cells than did ceftiofur (0.2–1.5 log_10_, 27.9–96.0%, kill, *p* < 0.0001 for all comparisons), enrofloxacin (1.1–3.9 log_10_, 82.2–99.9% kill, *p* values 0.0173–<0.0001), florfenicol (growth-0.9 log_10_, growth to 88.1% kill, *p* < 0.0001 for all comparisons), marbofloxacin (0.8–2.1 log_10_, 45.9–94.5% kill, *p* = 0.0011–<0.0001), tildipirosin (growth to 0.24 log_10_, growth to 26.4% kill, *p* < 0.0001 for all comparisons), tilmicosin (growth to 0.6 log_10_, growth to 68.4% kill, *p* < 0.0001 for all comparisons) and tulathromycin (growth to 0.3 log_10_, growth to 4% kill, *p* < 0.0001 for all comparisons). Following 2, 4, 6, 12 and 24 h after drug exposure, enrofloxacin killed more cells than did tilmicosin (*p* = 0.0210–<0.0001) and following 6, 12 and 24 h (*p* values 0.0285–<0.0001) more cells than tildipirosin; following 12 and 24 h enrofloxacin killed more cells than tulathromycin (*p* values 0.0185 and 0.0009, respectively).

In considering time, statistically significant differences were seen for treatment (*p* < 0.0001 for all comparisons), time (*p* < 0.0001 for all comparisons), and treatment by time (*p* < 0.0001 for all comparisons) for the 10^6^, 10^7^, 10^8^ and 10^9^ cfu/mL exposed to the C_max_ drug concentrations.

#### 3.1.4. Exposure to Maximum Tissue Total Drug Concentration (T_issuemax_)

Exposure of 10^6^ cfu/mL of *M. haemolytica* to the maximum tissue drug concentration yielded the following observations (Figure 4A). Pradofloxacin killed 99.99% of cells following 30 min of drug exposure and 100% of cells at 1 h and all other time points following drug exposure. Danofloxacin killed 87.5% of cells at 30 min, which increased to 99.9–100% of cells killed following 6 and 12 h of drug exposure, respectively. Enrofloxacin killed 98.9% of cells following 30 min, 99.99% following 2 h and 100% of cells following 6 h of drug exposure. Ceftiofur killed 16.7% of cells following 30 min, 95.3% of cells following 4 h and 100% of cells following 24 h of drug exposure. Florfenicol killed 14.9% of cells following 1 h, 94.5% after 4 h and 99.8% of cells following 24 h of drug exposure. Tildipirosin killed 20.8% of cells after 2 h and 99.9% of cells following 24 h of drug exposure. Tulathromycin killed 50.2% of cells after 1 h, 99.8% after 4 h and 99.99% of cells following 24 h of drug exposure.

Following exposure of 10^7^ cfu/mL of *M. haemolytica* to the maximum tissue drug concentration, pradofloxacin killed 99.99% of cells following 30 min and 1 h of drug exposure and 100% of cells at all time points thereafter (Figure 4B). Danofloxacin killed 90.9% of cells following 30 min, which increased to 99% of cells following 6 h and 99.99% and 100% of cells killed by 12 and 24 h after drug exposure, respectively. Enrofloxacin killed 97.8% of cells after 30 min, 99.99% after 4 and 6 h and 100% of cells at 12 and 24 h after drug exposure. Ceftiofur killed 49.7% of cells following 1 h, 99.0% after 6 h and 99.9% of cells following 24 h of drug exposure. Florfenicol killed 67.7% and 95.2% of cells following 4 and 24 h of drug exposure as compared to 29.6% and 71.3% for tildipirosin and 89.3 and 99.7% for tulathromycin, respectively.

Following exposure of 10^8^ cfu/mL of *M. haemolytica* to the maximum tissue drug concentration, pradofloxacin killed 99.99% of cells following 30 min to 12 h and 100% following 24 h of drug exposure (Figure 4C). Danofloxacin killed 92.8–98.6% of cells following 30 min to 4 h of drug exposure, which increased to 99.9% following 12 h of drug exposure. Enrofloxacin killed 97.1% of cells following 30 min, 99.9% following 6 h and 99.99% following 24 h of drug exposure. Ceftiofur killed 25.7% of cells following 30 min, 91.6% following 12 h and 79.9% following 24 h of drug exposure. Florfenicol killed 13.8% of cells following 1 h of drug exposure, but regrowth occurred at all time points thereafter. Tildipirosin and tulathromycin killed 21.7–27.2% of cells following 6 h and 65.4–70.7% of cells following 24 h of drug exposure.

Exposure of 10^9^ cfu/mL of *M. haemolytica* to the maximum tissue drug concentration yielded the following results (Figure 4D). Pradofloxacin killed 92.1% of cells following 30 min of drug exposure, 99.9% after 2 h, 99.99% after 6 and 12 h and 100% after 24 h of drug exposure. Danofloxacin killed 90.5% of cells after 30 min, 95.6–97.2% of cells following 4–12 h and 99.6% following 24 h of drug exposure. Enrofloxacin killed 87.7% of cells following 30 min, 99.8% following 4 h and 99.9% following 24 h of drug exposure. Ceftiofur, florfenicol, tildipirosin and tulathromycin killed between 49.3 and 88.2% of cells following 24 h of drug exposure. Following 1 h of drug exposure, pradofloxacin (2.3 log_10_, 99.5% kill) killed more cells than did tildipirosin (0.041 log_10_, 7.5% kill, *p* = 0.0413). Following 2, 4, 6, 12 and 24 h after drug exposure, pradofloxacin (3.2–9.1 log_10_, 99.9–100% kill) killed more cells than did ceftiofur (0.1–1.0 log_10_, 9.4–88.2% kill, *p* values 0.0013–<0.0001), florfenicol (growth-0.3 log_10_, growth-49.3% kill, *p* values 0.0086–<0.0001), tildipirosin (growth-0.9 log_10_, growth-85.5% kill, *p* values < 0.0001 for all comparisons) and tulathromycin (0.1–2.3 log_10_, 25.7–88.1% kill, *p* values 0.0030–<0.0001). Following 24 h of drug exposure, pradofloxacin (9.1 log_10_, 100% kill) killed more cells than did enrofloxacin (4.3 log_10_, 99.9% kill, *p* < 0.0001). Following 2, 4, 6 and 12 h of drug exposure, enrofloxacin (2.5–3.4 log_10_, 99.3–98.3% kill) killed more cells than did ceftiofur (0.1–0.2 log_10_, 9.4–31.2% kill, *p* values 0.0056–<0.0001), florfenicol (growth; *p* values 0.0106– < 0.0001), tildipirosin (growth-0.1 log_10_, growth-8.9% kill, *p* values 0.0014–<0.0001) and tulathromycin (0.1–0.2 log_10_, growth to 28% kill, *p* values 0.0112–<0.0001). Following 24 h of drug exposure, enrofloxacin (4.3 log_10_, 99.9% kill) killed more cells than did tildipirosin (0.8 log_10_, 85.5% kill, *p* < 0.0001).

Regarding time, statistically significant differences were seen for treatment (*p* < 0.0001 for all comparisons), time (*p* < 0.0001) and treatment by time (*p* = 0.0042– < 0.0001) for the 10^6^, 10^7^, 10^8^ and 10^9^ cfu/mL exposed to the maximum tissue drug concentrations.

### 3.2. Pasteurella multocida

#### 3.2.1. Exposure to Minimum Inhibitory Drug Concentration (MIC)

**Figure 5:**
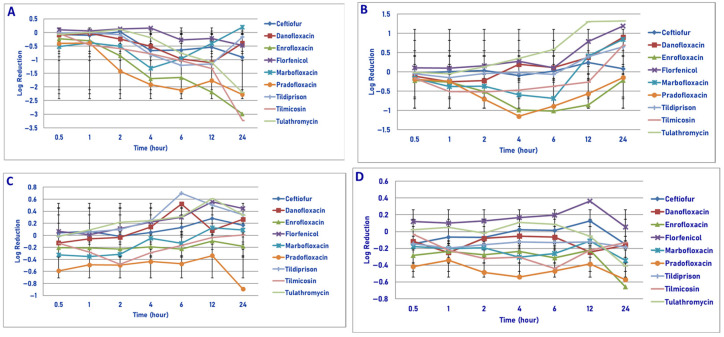
Comparative killing of *Pasteurella multocida* by 9 antimicrobial agents at the MIC: (**A**) 10^6^ cfu/mL, (**B**) 10^7^cfu/mL, (**C**) 10^8^ cfu/mL, (**D**) 10^9^ cfu/mL drug concentrations. (**A**) MIC 10^6^ cfu/mL. twenty-four hours: danofloxacin vs. enrofloxacin (*p* = 0.0078), tilmicosin (*p* < 0.0001); enrofloxacin vs. ceftiofur (*p* = 0.0120); florfenicol (*p* = 0.0163), tildipirosin (*p* < 0.0001); ceftiofur vs. tilmicosin (*p* < 0.0001); marbofloxacin vs. tilmicosin (*p* < 0.0001), tulathromycin (*p* < 0.0001); pradofloxacin vs. tildipirosin (*p* = 0.0164); tilmicosin vs. tildipirosin (*p* < 0.0001); tulathromycin vs. tildipirosin (*p* = 0.0012). (**B**) MIC 10^7^ cfu/mL. Six hours: pradofloxacin vs. ceftiofur (*p* < 0.0001), danofloxacin (*p* < 0.0001), florfenicol (*p* < 0.0001), marbofloxacin (*p* = 0.0001), tildipirosin (*p* < 0.0001), tilmicosin *p* = 0.0001), tulathromycin (*p* < 0.0001); enrofloxacin vs. pradofloxacin (*p* = 0.0205). (**C**) MIC 10^8^ cfu/mL. No statistical significant differences. (**D**) MIC 10^9^ cfu/mL. Four hours: pradofloxacin vs. ceftiofur (*p* = 0.0012), danofloxacin (*p* = 0.007), enrofloxacin (*p* = 0.0020), florfenicol (*p* = 0.0015), marbofloxacin (*p* = 0.0278), tildipirosin (*p* = 0.0058), tilmicosin (*p* = 0.0281), tulathromycin (*p* = 0.0022).

**Figure 6:**
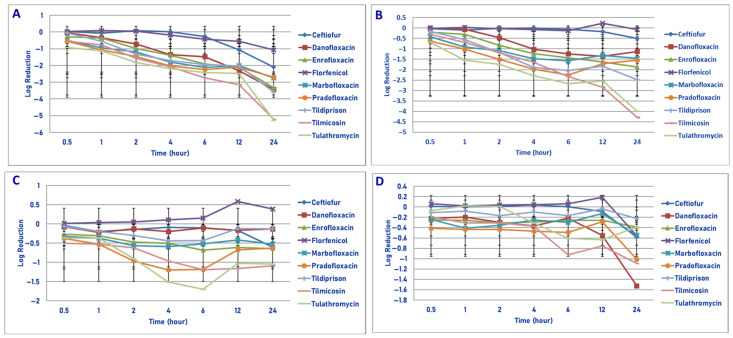
Comparative killing of *Pasteurella multocida* by 9 antimicrobial agents at the MPC: (**A**) 10^6^ cfu/mL, (**B**) 10^7^ cfu/mL, (**C**) 10^8^ cfu/mL, (**D**) 10^9^ cfu/mL drug concentrations. (**A**) MPC 10^6^ cfu/mL. Two hours: tildipirosin vs. ceftiofur (*p* = 0.0197); tilmicosin vs. ceftiofur (*p* = 0.0003); tulathromycin vs. ceftiofur (*p* < 0.0001); tilmicosin vs. florfenicol (*p* = 0.0027); tulathromycin vs. ceftiofur (*p* < 0.0001); pradofloxacin vs. ceftiofur (*p* = 0.0368). Four hours: enrofloxacin vs. ceftiofur (*p* = 0.0434); marbofloxacin vs. florfenicol (*p* = 0.0223); pradofloxacin vs. ceftiofur (*p* < 0.0001), florfenicol (*p* = 0.0078); tildipirosin vs. ceftiofur (*p* = 0.0003), florfenicol (*p* = 0.0131); tilmicosin vs. ceftiofur (*p* < 0.0001), florfenicol (*p* < 0.0001); tulathromycin vs. ceftiofur (*p* < 0.0001), florfenicol (*p* < 0.0001). Six hours: danofloxacin vs. tilmicosin (*p* = 0.0314); enrofloxacin vs. ceftiofur (*p* = 0.0019); marbofloxacin vs. ceftiofur (*p* = 0.0001), florfenicol (*p* = 0.0077); pradofloxacin vs. ceftiofur (*p* = 0.0002), florfenicol (*p* = 0.0105); tildipirosin vs. ceftiofur (*p* = 0.0006), florfenicol (*p* = 0.0168); tilmicosin vs. ceftiofur (*p* < 0.0001), florfenicol (*p* < 0.0001); tulathromycin vs. ceftiofur (*p* < 0.0001), florfenicol (*p* = 0.0006). Twelve hours: danofloxacin vs. florfenicol L (*p* = 0.0175); enrofloxacin vs. ceftiofur (*p* = 0.0553), florfenicol (*p* = 0.0225); tildipirosin vs. florfenicol (*p* = 0.0248); tilmicosin vs. ceftiofur (*p* < 0.0001), florfenicol (*p* < 0.0001); tulathromycin vs. ceftiofur (*p* = 0.000), florfenicol (*p* < 0.0001). Twenty-four hours: danofloxacin vs. ceftiofur (*p* < 0.0001), florfenicol (*p* < 0.0002), tilmicosin (*p* < 0.0001) and tulathromycin (*p* < 0.0001); enrofloxacin vs. ceftiofur (*p* < 0.0001), florfenicol (*p* < 0.0001); tildipirosin vs. ceftiofur (*p* < 0.0001), florfenicol (*p* < 0.0001); tilmicosin vs. ceftiofur (*p* < 0.0001), florfenicol (*p* < 0.0001), enrofloxacin (*p* < 0.0001), pradofloxacin (*p* < 0.0001); tulathromycin vs. ceftiofur (*p* < 0.0001), florfenicol (*p* < 0.0001), enrofloxacin (*p* < 0.0001), pradofloxacin (*p* < 0.0001); tilmicosin vs. tildipirosin (*p* = 0.0012); tulathromycin vs. tildipirosin (*p* = 0.0003). (**B**) MPC 10^7^ cfu/mL. One hour: danofloxacin vs. tulathromycin (*p* < 0.0001), tulathromycin vs. ceftiofur (*p* = 0.0002); enrofloxacin (*p* = 0.0316), florfenicol (*p* < 0.0001). Two hours: danofloxacin vs. tulathromycin (*p* = 0.0003); tilmicosin vs. ceftiofur (*p* = 0.0081), florfenicol (*p* = 0.0341); tulathromycin vs. ceftiofur (*p* < 0.0001), florfenicol (*p* < 0.0001). Four hours: danofloxacin vs. tulathromycin (*p* = 0.0004); enrofloxacin vs. ceftiofur (*p* = 0.0295); marbofloxacin vs. ceftiofur (*p* = 0.0006), florfenicol (*p* = 0.0073); pradofloxacin vs. ceftiofur (*p* = 0.0106); tildipirosin vs. ceftiofur (*p* < 0.0001), florfenicol (*p* < 0.0001); tilmicosin vs. ceftiofur (*p* < 0.0001), florfenicol (*p* = 0.0001); tulathromycin vs. ceftiofur (*p* < 0.0001), florfenicol (*p* < 0.00010). Six hours: danofloxacin vs. tilmicosin (*p* = 0.0051), tulathromycin (*p* < 0.0001); enrofloxacin vs. ceftiofur (*p* = 0.0033); marbofloxacin vs. ceftiofur (*p* = 0.0003); pradofloxacin vs. ceftiofur (*p* < 0.0001), florfenicol 0.0034); tildipirosin vs. ceftiofur (*p* < 0.0001), florfenicol (*p* = 0.0013); tilmicosin vs. ceftiofur (*p* < 0.0001), florfenicol (*p* < 0.0001); tulathromycin vs. ceftiofur (*p* < 0.0001), enrofloxacin (*p* = 0.0064), florfenicol (*p* < 0.0001), marbofloxacin (*p* = 0.0524). Twelve hours: danofloxacin vs. tilmicosin (*p* < 0.0001), tulathromycin (*p* = 0.0014); enrofloxacin vs. ceftiofur (*p* = 0.0011), florfenicol (*p* = 0.0013); pradofloxacin vs. ceftiofur (*p* = 0.0420), florfenicol (*p* = 0.0485); tildipirosin vs. ceftiofur (*p* = 0.0001), florfenicol (*p* = 0.0002); tilmicosin vs. ceftiofur (*p* < 0.0001), florfenicol (*p* < 0.0001), enrofloxacin (*p* = 0.0084), pradofloxacin (*p* = 0.0002); tulathromycin vs. ceftiofur (*p* < 0.0001), florfenicol (*p* < 0.0001), marbofloxacin (*p* = 0.0086), pradofloxacin (*p* = 0.0197). Twenty-four hours: danofloxacin vs. tildipirosin (*p* = 0.0006), tilmicosin (*p* < 0.0001), tulathromycin (*p* < 0.0001); enrofloxacin vs. ceftiofur (*p* = 0.0041), florfenicol (*p* = 0.0037); tildipirosin vs. ceftiofur (*p* < 0.0001), florfenicol (*p* < 0.0001); tilmicosin vs. ceftiofur (*p* < 0.0001), florfenicol (*p* < 0.0001), enrofloxacin *p* < 0.0001), marbofloxacin (*p* < 0.0001), pradofloxacin (*p* < 0.0001), tildipirosin (*p* < 0.0001); tulathromycin vs. ceftiofur (*p* < 0.0001), florfenicol (*p* < 0.0001), enrofloxacin (*p* < 0.0001), marbofloxacin (*p* < 0.0001), pradofloxacin (*p* < 0.0001), tildipirosin (*p* < 0.0001). (**C**) MPC 10^8^ cfu/mL. Six hours: danofloxacin vs. tulathromycin (*p* = 0.0089); tulathromycin vs. ceftiofur (*p* = 0.0227), florfenicol (*p* = 0.0029). (**D**) MPC 10^9^ cfu/mL. No statistically significant observations.

**Figure 7:**
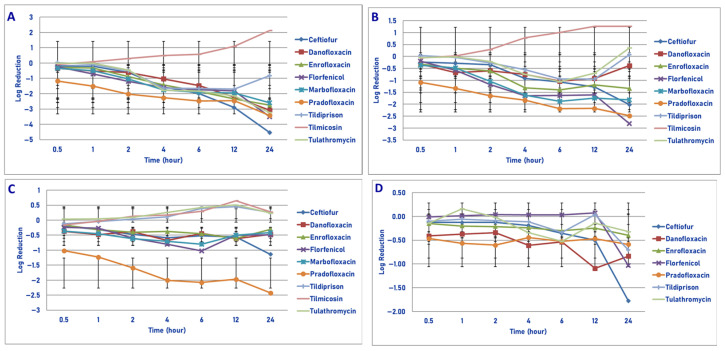
Comparative killing of *Pasteurella multocida* by 9 antimicrobial agents at the C_max_: (**A**) 10^6^ cfu/mL, (**B**) 10^7^ cfu/mL, (**C**) 10^8^ cfu/mL, (**D**) 10^9^ cfu/mL drug concentrations. (**A**) C_max_ 10^6^ cfu/mL. Two hours: florfenicol vs. tilmicosin (*p* = 0.0333); pradofloxacin vs. tilmicosin (*p* = 0.0004). Four hours: ceftiofur vs. tilmicosin (*p* = 0.0002); enrofloxacin vs. tilmicosin (*p* = 0.0079); florfenicol vs. tilmicosin (*p* < 0.0001); marbofloxacin vs. tilmicosin (*p* < 0.0001); pradofloxacin vs. tilmicosin (*p* < 0.0001); tildipirosin vs. tilmicosin (*p* < 0.0001); tulathromycin vs. tilmicosin (*p* < 0.0001). Six hours: ceftiofur vs. tilmicosin (*p* < 0.0001); enrofloxacin vs. tilmicosin (*p* < 0.0001); florfenicol vs. tilmicosin (*p* < 0.0001); marbofloxacin vs. tilmicosin (*p* < 0.0001); pradofloxacin vs. tilmicosin (*p* < 0.0001); tildipirosin vs. tilmicosin (*p* < 0.0001), tulathromycin vs. tilmicosin (*p* < 0.0001). Twelve hours: ceftiofur vs. tilmicosin (*p* < 0.0001); enrofloxacin vs. tilmicosin (*p* < 0.0001); florfenicol vs. tilmicosin (*p* < 0.0001); marbofloxacin vs. tilmicosin (*p* < 0.0001); pradofloxacin vs. tilmicosin (*p* < 0.0001); tildipirosin vs. tilmicosin (*p* < 0.0001); tulathromycin vs. tilmicosin (*p* < 0.0001). Twenty-four hours: ceftiofur vs. enrofloxacin (*p* = 0.0021), marbofloxacin (*p* = 0.0004), tildipirosin (*p* < 0.0001), tilmicosin (*p* < 0.0001); enrofloxacin vs. tildipirosin (*p* = 0.0009), tilmicosin (*p* < 0.0001); florfenicol vs. tildipirosin (*p* = 0.0004), tilmicosin (*p* < 0.0001); marbofloxacin vs. tildipirosin (*p* = 0.0004), tilmicosin (*p* < 0.0001); pradofloxacin vs. tildipirosin (*p* < 0.0001), tilmicosin (*p* < 0.0001); tilmicosin vs. tildipirosin (*p* < 0.0001); tulathromycin vs. tildipirosin (*p* < 0.0001), tilmicosin (*p* < 0.0001). (**B**) C_max_ 10^7^ cfu/mL. Two hours: florfenicol vs. tilmicosin (*p* = 0.0195); pradofloxacin vs. tilmicosin (*p* = 0.0046). Four hours: enrofloxacin vs. tilmicosin (*p* = 0.0228); florfenicol vs. tilmicosin (*p* < 0.0001); marbofloxacin vs. tilmicosin (*p* < 0.0001); pradofloxacin vs. tilmicosin (*p* < 0.0001). Six hours: ceftiofur vs. tilmicosin (*p* = 0.0022); enrofloxacin vs. tilmicosin (*p* = 0.0006); florfenicol vs. tilmicosin (*p* < 0.0001); marbofloxacin vs. tilmicosin (*p* < 0.0001); pradofloxacin vs. tilmicosin (*p* < 0.0001); tildipirosin vs. tilmicosin (*p* = 0.0067); tulathromycin vs. tilmicosin (*p* = 0.0003). Twelve hours: ceftiofur vs. tilmicosin (*p* < 0.0001); enrofloxacin vs. tilmicosin (*p* = 0.0003); florfenicol vs. tilmicosin (*p* < 0.0001); marbofloxacin vs. tilmicosin (*p* < 0.0001); pradofloxacin vs. tilmicosin (*p* < 0.0001); tildipirosin vs. tilmicosin (*p* = 0.0003); tulathromycin vs. tilmicosin (*p* = 0.0012). Twenty-four hours: ceftiofur vs. tilmicosin (*p* < 0.0001), tildipirosin (*p* = 0.0008), tulathromycin (*p* = 0.0004); florfenicol vs. enrofloxacin (*p* = 0.0066), tildipirosin (*p* < 0.0001), tilmicosin (*p* < 0.0001), tulathromycin (*p* < 0.0001); enrofloxacin vs. tilmicosin (*p* < 0.0001); marbofloxacin vs. tildipirosin (*p* = 0.0046), tilmicosin (*p* < 0.0001), tulathromycin (*p* = 0.0010); pradofloxacin vs. tildipirosin (*p* < 0.0001), tilmicosin (*p* < 0.0001), tulathromycin (*p* < 0.0001). (**C**) C_max_ 10^8^ cfu/mL. Two hours: pradofloxacin vs. tildipirosin (*p* = 0.0420), tilmicosin (*p* = 0.0125), tulathromycin (*p* = 0.0490). Four hours: pradofloxacin vs. enrofloxacin (*p* = 0.0188), tildipirosin (*p* < 0.0001), tilmicosin (*p* < 0.0001), tulathromycin (*p* < 0.0001). Six hours: pradofloxacin vs. ceftiofur (*p* = 0.0320), enrofloxacin (*p* = 0.0190), tildipirosin (*p* < 0.0001), tilmicosin (*p* < 0.0001), tulathromycin (*p* < 0.0001); florfenicol vs. tildipirosin (*p* = 0.0033), tilmicosin (*p* = 0.0163), tulathromycin (*p* = 0.0051). Twelve hours: pradofloxacin vs. tildipirosin (*p* < 0.0001); tilmicosin (*p* < 0.0001), tulathromycin (*p* < 0.0001). Twenty-four hours: ceftiofur vs. tildipirosin (*p* = 0.0292), tilmicosin (*p* = 0.0305); pradofloxacin vs. enrofloxacin (*p* < 0.0001), florfenicol *p* = 0.0008), marbofloxacin (*p* = 0.0003), tildipirosin (*p* < 0.0001), tilmicosin (*p* < 0.0001), tulathromycin (*p* < 0.0001). (**D**) C_max_ 10^9^ cfu/mL. Twenty-four hours: ceftiofur vs. tildipirosin (*p* = 0.0143), tilmicosin (*p* = 0.0043), tulathromycin (*p* = 0.0187).

**Figure 8:**
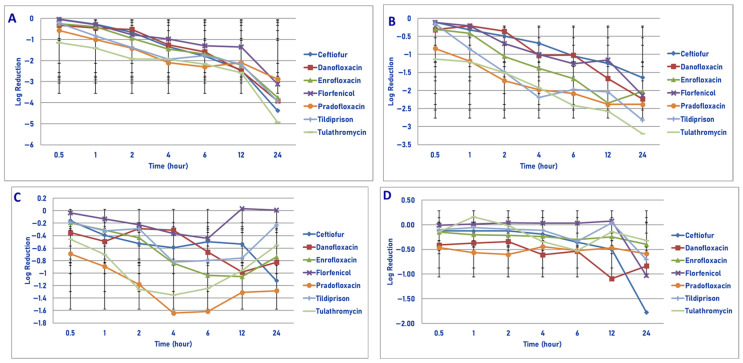
Comparative killing of *Pasteurella multocida* by 6 antimicrobial agents at the T_issuemax_: (**A**) 10^6^ cfu/mL, (**B**) 10^7^ cfu/mL, (**C**) 10^8^ cfu/mL, (**D**) 10^9^ cfu/mL drug concentrations. (**A**) T_issuemax_ 10^6^ cfu/mL. Six hours: danofloxacin vs. tilmicosin (*p* = 0.0001); twelve hours: danofloxacin vs. tilmicosin (*p* < 0.0001); twenty-four hours: danofloxacin vs. tildipirosin (*p* < 0.0001), tilmicosin (*p* < 0.0001). Ceftiofur vs. pradofloxacin (*p* = 0.0197); tulathromycin vs. florfenicol (*p* = 0.0126), pradofloxacin (*p* < 0.0001). (**B**) T_issuemax_ 10^7^ cfu/mL. Six hours: danofloxacin vs. tilmicosin (*p* = 0.0315). Twelve hours: danofloxacin vs. tilmicosin (*p* = 0.0001). Twenty-four hours: danofloxacin vs. florfenicol (*p* < 0.0001), pradofloxacin vs. danofloxacin (*p* = 0.0057). (**C**) T_issuemax_ 10^8^ cfu/mL. Twenty-four hours: pradofloxacin vs. danofloxacin (*p* = 0.0076). (**D**) T_issuemax_ 10^9^ cfu/mL. Twenty-four hours: ceftiofur vs. enrofloxacin (*p* < 0.0001), pradofloxacin (*p* < 0.0001), tildipirosin (*p* = 0.0226), tulathromycin (*p* = 0.0003).

Exposure of 10^6^ cfu/mL of *P. multocida* to the MIC drug concentration did not result in any significant differences in killing between the agents at 0.5, 1, 2, 4, 6 and 12 h after drug exposure (Figure 5A). Pradofloxacin killed 59.2% of cells following 30 min, 98.3% after 4 h and 98.9% following 24 h of drug exposure. Ceftiofur killed up to 56.4% of cells following 6 h, of drug exposure but regrowth occurred thereafter. Danofloxacin killed up to 80% of cells following 6 h of drug exposure, but regrowth occurred thereafter. Enrofloxacin killed 46.6% of cells after 30 min, 97.9% after 4 h and 99.8% following 24 h of drug exposure. Marbofloxacin killed up to 91.9% of cells following 4 h of drug exposure, but regrowth occurred thereafter. Florfenicol killed no more than 10.9% of cells at any time point and regrowth occurred. Tildipirosin killed up to 92.6% of cells following 6 h of drug exposure; however, regrowth occurred thereafter. Tulathromycin killed 89.7% of cells following 12 h of drug exposure and 83.1% following 24 h of drug exposure. Tilmicosin killed 82.2% of cells following 4 h and 99.9% of cells following 24 h of drug exposure. Statistically significant differences in kill between drugs and at the various drug concentrations tested are highlighted below each figure (Figure 5, Figure 6, Figure 7 and Figure 8). Regarding time, statistically significant differences were seen for treatment (*p* = 0.0104), time (*p* < 0.0001) and treatment by time (*p* < 0.0001).

Following exposure of 10^7^ cfu/mL of *P. multocida* to the MIC drug concentration, significant differences in killing between the drugs was not seen following 0.5, 1, 2, 4, 12 and 24 h of drug exposure (Figure 5B). Pradofloxacin killed up to 87.1% of cells following 4 h of drug exposure, which decreased to 69.5 and 11.5% kill following 12 and 24 h of drug exposure. Danofloxacin killed up to 33.3% of cells by 2 h, after which regrowth occurred. Enrofloxacin killed up to 90.4% of cells by 6 h and 40.1% of cells following 24 h of drug exposure. Marbofloxacin killed up to 63.1% of cells following 4 h of drug exposure, but regrowth occurred thereafter. Initial killing in the presence of tildipirosin, tilmicosin and tulathromycin (6.2–63.4% kill) following 1 h of drug exposure was followed by regrowth. No killing occurred in the presence of florfenicol at any time point. For time, significant differences were seen for treatment (*p* = 0.0002), time (*p* < 0.0001) and treatment by time (*p* = 0.0141).

Following exposure of 10^8^ cfu/mL of *P. multocida* to the MIC drug concentration, significant differences were not seen at any time point following drug exposure (Figure 5C). Pradofloxacin killed between 50.9 and 67.8% of cells following cell time of drug exposure as compared to between 19.6 and 40.1% for enrofloxacin. Danofloxacin killed 22.9% of cells following 30 min of drug exposure, but regrowth occurred thereafter. Ceftiofur, florfenicol, tildipirosin and tulathromycin had growth at all time points. Marbofloxacin killed up to 45.4% of cells at 30 min following drug exposure, which declined thereafter and regrowth occurred. Tilmicosin killed up to 56.4% of cells (2 h) but declined thereafter and regrowth occurred. For time, a statistically significant difference was seen (*p* = 0.003).

Following exposure of 10^9^ cfu/mL of *P. multocida* to the MIC drug concentration, significant differences in killing between the drugs was only seen following 4 h of drug exposure (Figure 5D). Pradofloxacin (0.5 log_10_, 70.2% kill) killed more cells than did ceftiofur (growth, *p* = 0.0012), enrofloxacin (0.2 log_10_, 40.6% kill, *p* = 0.0020), florfenicol (growth, *p* = 0.0015), marbofloxacin (0.3 log_10_, 41.3% kill, *p* = 0.278), tildipirosin (0.1 log_10_, 47.3% kill, *p* = 0.0058), tilmicosin (0.3 log_10_, 47.3% kill, *p* = 0.0281) and tulathromycin (growth, *p* = 0.0022). Pradofloxacin killed between 61.4 and 73.2% of cells, danofloxacin between 8.1 and 33.1%, enrofloxacin between 35.9 and 71.7% and marbofloxacin between 8.8 and 41.9% of cells following drug exposure at all time points. For the other agents, killing was as follows following 24 h of drug exposure: ceftiofur (1.6% kill), florfenicol (growth), tildipirosin (16.4% kill), tilmicosin (22.5% kill) and tulathromycin (57.2% kill).

#### 3.2.2. Exposure to Mutant Prevention Drug Concentration (MPC)

Exposure of 10^6^ cfu/mL of *P. multocida* to the MPC drug concentration of the 9 agents tested yielded the following results (Figure 6A). Pradofloxacin killed 71.9% of cells following 30 min, 99.1% after 4 h and 99.7% after 24 h of drug exposure. By comparison, enrofloxacin and marbofloxacin killed 47.6% and 65.1%, 98.7% and 99.2% and 99.9% and 99.8% of cells following 30 min, 6 h and 24 h of drug exposure, respectively. Danofloxacin killed 7.6% of cells following 30 min, 95.2% following 4 h and 99.9% following 24 h of drug exposure. Ceftiofur killed 44.9% and 98.7% of cells following 6 and 24 h of drug exposure. Florfenicol killed 71.4% and 84.3% of cells following 12 and 24 h of drug exposure. Tildipirosin, tilmicosin and tulathromycin killed between 98.7 and 99.8% of cells and between 99.9 and 9.99% of cells following 6 and 24 h of drug exposure.

Exposure of 10^7^ cfu/mL of *P. multocida* to the MPC drug concentration resulted in more cells being killed by tulathromycin (1.6 log_10_, 96.9% kill) than by ceftiofur (0.01 log_10_, 19.5% kill, *p* = 0.0002) and enrofloxacin (0.3 log_10_, 48.9% kill, *p* = 0.0316) following 1 h of drug exposure (Figure 6B). Pradofloxacin killed 74.6% of cells following 30 min of drug exposure, which increased to 98.6% following 24 h of drug exposure. Danofloxacin killed between 9.8 and 58.1% of cells following 1–2 h, 95.6% following 12 h and 91.6% following 24 h of drug exposure. Enrofloxacin and marbofloxacin killed between 36.9 and 59.9% of cells following 30 min of drug exposure, which increased to between 94.6 and 98.6% kill following 24 h of drug exposure. Florfenicol killed up to 9.9% of cells following 4 h of drug exposure, but regrowth occurred thereafter. Tildipirosin, tilmicosin and tulathromycin killed between 30.1 and 58.8% of cells following 30 min of drug exposure, between 8.4 and 99.7% after 6 h and between 99.6 and 99.99% after 24 h of drug exposure.

Exposure of 10^8^ cfu/mL of *P. multocida* to the MPC drug concentrations of the 9 drugs tested gave the following results (Figure 6C). Pradofloxacin killed between 57.5% of cells following 30 min of drug exposure, 93.2% after 6 h and 73.9% of cells following 24 h of drug exposure. By comparison, enrofloxacin and marbofloxacin killed between 46.3 and 51.4% of cells following 30 min, between 64.0 and 77.2% of cells after 6 h and between 63.7 and 75.8% of cells following 24 h of drug exposure. Danofloxacin killed between 10.9 and 35.4% of cells over all time points of drug exposure. Ceftiofur killed 29.3% of cells after 1 h, 13.0% after 6 h and 71.8% following 24 h of drug exposure. Florfenicol had growth at all time points. Tildipirosin killed 62.2% of cells after 4 h and 20.4% of cells following 24 h of drug exposure. Tilmicosin killed 55.0% of cells after 30 min, 73.5% of cells after 6 h and 58.6% of cells following 24 h of drug exposure. Tulathromycin killed 48.5% of cells after 30 min, 96.5% of cells after 4 h and 81.9% of cells following 24 h of drug exposure. Following 6 h of drug exposure, more cells were killed by tulathromycin (1.7 log_10_, 89.8% kill) than by ceftiofur (0.1 log_10_, 13.0% kill, *p* = 0.0227) or florfenicol (growth, *p* = 0.0029). Regarding time, statistically significant differences were seen for treatment (*p* = 0.0020–<0.0002), time (*p* = 0.0008–<0.0001) and treatment by time (*p* ≤ 0.0001) for the 10^6^, 10^7^ and 10^8^ cfu/mL densities exposed to the MPC drug concentrations.

Exposure of 10^9^ cfu/mL of *P. multocida* to the MPC drug concentration did not result in any statistical differences in kill between the various agents tested and at any time points (Figure 6D). Pradofloxacin killed 59.7% of cells following 30 min of drug exposure, which increased to 70.3% kill following 24 h of drug exposure. Danofloxacin killed 38.1–71.3% of cells following 30 min-12 h of drug exposure and 92.8% following 24 h of drug exposure. By comparison, the remaining agents’ kill rates at the same time points, respectively, were as follows: ceftiofur growth and 76.1% kill, enrofloxacin 43.5% and 56.9% kill, florfenicol growth and 38.4% kill, marbofloxacin 20.1% and 62.6% kill, tildipirosin 12.0% and 37.8% kill, tilmicosin 28.9% and 81.2% kill and tulathromycin 13.8% and 51.6% kill. At 10^9^ cfu/mL, a statistically significant difference was seen for time (*p* < 0.0001) but not for treatment nor treatment by time.

#### 3.2.3. Exposure to Maximum Serum Total Drug Concentration (C_max_)

Following exposure of 10^6^ cfu/mL of *P. multocida* to the maximum serum drug concentration of each agent, a significant difference was seen between florfenicol (1.2 log_10_, 92.9% kill) and tilmicosin (growth, *p* = 0.0333). Pradofloxacin (1.5 log_10_, 96.7% kill) killed more cells than did tilmicosin (*p* = 0.0004). Pradofloxacin killed 92.7% of cells following 30 min of drug exposure, 99.3% after 2 h and 99.9% after 24 h of drug exposure (Figure 7A). Danofloxacin killed 43.4% of cells after 30 min, 90.1–96.4% after 4–6 h and 99.9% after 24 h of drug exposure. Enrofloxacin and marbofloxacin killed 47.1–51.9% of cells after 30 min, 85.2–90.2% kill after 2 h and 99.7–99.8% kill after 24 h of drug exposure. Ceftiofur, florfenicol, tildipirosin and tulathromycin killed the following percentage of cells following 30 min, 2 h and 24 h of drug exposure: ceftiofur 34.6%, 58.0%, 99.99%; florfenicol 35.4%, 92.9%, 99.9%; tildipirosin growth, 56.1%, 69.8%; tulathromycin 1%, 59.3%, 99.2%. Tilmicosin killed 8.9% of cells following 30 min of drug exposure but had growth at every time point thereafter.

Following exposure of 10^7^ cfu/mL of *P. multocida* to the C_max_ drug concentration, pradofloxacin killed 91.5% of cells following 30 min of drug exposure, which increased to 99.3% following 6 h and 99.6% following 24 h of drug exposure (Figure 7B). Danofloxacin killed 58.4–81.2% of cells following 1–6 h, 59.6% at 12 h and had growth at 24 h following drug exposure. Enrofloxacin and marbofloxacin killed between 50.6% and 54.4% of cells after 30 min, between 95.9 and 98.1% after 6 h and between 94.3 and 97.9% following 24 h of drug exposure. Ceftiofur and florfenicol killed 34.1–35.7%, 91.4–97.2% and 98.2–99.6% of cells following 30 min, 6 h and 24 h of drug exposure. Tildipirosin and tulathromycin killed up to 88.1–90.2% of cells following 6 h of drug exposure, but growth occurred thereafter. Tilmicosin killed 7.1% of cells following 30 min of drug exposure but had growth at all time points thereafter.

Exposure of 10^8^ cfu/mL of *P. multocida* to the maximum serum drug concentration yielded the following results (Figure 7C). Pradofloxacin killed 90.4% of cells after 30 min of drug exposure, which increased to 99.2% after 6 h and 97.6% following 24 h of drug exposure. Danofloxacin killed between 31.5 and 46.4% of cells after 30 min-4 h, 31.5% after 6 h and 17.8 and 16.2%, respectively, after 12 and 24 h of drug exposure. Enrofloxacin and marbofloxacin killed the following percentages of cells following 30 min, 6 h and 24 h of drug exposure: 26.6% and 55.7%, 63.4% and 70.7% and 45.7% and 48.0%. Ceftiofur killed 35.1%, 67.7% and 92.7% of cells following 30 min, 6 h and 24 h of drug exposure. Florfenicol killed 39.3%, 87.8% and 59.1% of cells following 30 min, 6 h and 24 h of drug exposure. Tulathromycin had growth at all time points. Tildipirosin and tilmicosin killed up to 17.2% of cells in the first 30 min to 2 h following drug exposure, but growth occurred thereafter.

Regarding time, statistically significant differences were seen for treatment (*p* < 0.0001–0.0029), time (*p* < 0.0001–0.0001) and treatment by time (*p* < 0.0001 for all comparisons) for the 10^6^, 10^7^ and 10^8^ cfu/mL densities exposed to the maximum serum drug concentration.

Exposure of 10^9^ cfu/mL of *P. multocida* to the maximum serum drug concentration provided the following observations (Figure 7D). Pradofloxacin killed 67.4% of cells following 30 min of drug exposure, which increased to 84.4% and 83.4% following 4 h and 24 h of drug exposure. Following 30 min, 4 h and 24 h of drug exposure, ceftiofur, enrofloxacin and marbofloxacin killed the following percentages of cells, respectively: 4.5%, 22.7%, 75.5%; 46.8%, 50.3%, 58.9%; and 33.7%, 42.9%, 40.5%. Danofloxacin killed between 44.6 and 77.3% of cells over all time periods. Florfenicol killed 5.4% and 70.9% of cells following 4 and 24 h of drug exposure. Killing was minimal with tildipirosin, tilmicosin and tulathromycin, ranging from 10.7 to 22.7% kill following 24 h of drug exposure. Following 24 h of drug exposure, ceftiofur (1.4 log_10_, 75.5% kill) killed more bacteria than did tildipirosin (0.1 log_10_, 21.6% kill, *p* = 0.0143), tilmicosin (0.1 log_10_, 10.7% ill, *p* = 0.0143) and tulathromycin (0.1 log_10_, 22.7% kill, *p* = 0.0187).

At the 10^9^ cfu/mL density, a statistically significant difference was seen for time (*p* < 0.0001) and treatment by time (*p* = 0.0060).

#### 3.2.4. Exposure to Maximum Tissue Total Drug Concentration (T_issuemax_)

Following exposure of 10^6^ cfu/mL of *P. multocida* to the maximum tissue drug concentrations of the seven antimicrobials tested, pradofloxacin killed 72.4%, 99.5% and 99.9% of cells following 30 min, 6 h and 24 h of drug exposure (Figure 8A). Danofloxacin killed 49.8–69.6% of cells after 30 min to 2 h and 99.3–99.8% of cells following 12 and 24 h of drug exposure. For ceftiofur and florfenicol, 6.1–10.1%, 93.4–97.9% and 99.9–99.99% of cells were killed following 30 min, 6 h and 24 h of drug exposure. Tildipirosin killed 39.4% of cells following 30 min of drug exposure, which increased to 98.1% after 6 h and 99.9% after 24 h of drug exposure. Tulathromycin killed 90.5%, 99.0% and 99.99% of cells following 30 min, 6 h and 24 h of drug exposure.

A statistically significant difference was seen for treatment (*p* = 0.0031), time (*p* < 0.0001) and treatment by time (*p* < 0.0001).

Exposure of 10^7^ and 10^8^ cfu/mL of *P. multocida* to the maximum tissue drug concentration did not result in any significant differences in killing between the agents compared (Figure 8B,C). Pradofloxacin (10^7^ cfu/mL) and tulathromycin killed 85.2–90.4%, 99.2 and 99.2% and 99.6–99.9% of cells following 30 min, 6 h and 24 h of drug exposure. Danofloxacin killed between 34.1 and 49.4% and between 68.4 and 96.4% following 30 min, 6 h and 24 h of drug exposure, respectively. Ceftiofur and florfenicol killed 20.6–21.9%, 84.5–93.5% and 93.5–99.0% of cells following 30 min, 6 h and 24 h of drug exposure. For enrofloxacin, those values, respectively, were 47.2%, 97.5% and 98.8%. Tildipirosin killed 54.1%, 98.4% and 99.5% of cells following 30 min, 6 h and 24 h of drug exposure. Pradofloxacin (10^8^ cfu/mL) killed 78.4%, 95.8% and 89.0% of cells following 30 min, 4 h and 24 h of drug exposure. For the remaining drugs, those values, respectively, were as follows: ceftiofur 29.2%, 73.7%, 91.4%; enrofloxacin 35.3%, 82.9%, 74.2%; florfenicol 10.8%, 54.3%, growth; tildipirosin 31.9%, 66.9%, 27.7%; and tulathromycin 59.9%, 89.7%, 72.0%.

At the 10^7^ cfu/mL density, a statistically significant difference was seen for treatment (*p* = 0.0453) and time (*p* < 0.0001) and at the 10^8^ cfu/mL density for time (*p* = 0.0010).

Following exposure of 10^9^ cfu/mL of *P. multocida* to the maximum tissue concentration of the agents tested, pradofloxacin killed 63.4%, 67.9% and 70.7% of cells following 30 min, 6 h and 24 h after drug exposure (Figure 8D). Danofloxacin killed between 42.7 and 70.2% of cells following 30 min to 4 h, 59.4% following 6 h and 89.0% and 84.3% of cells following 12 and 24 h of drug exposure. For the remaining drugs, those values, respectively, were as follows: ceftiofur 24.1%, 50.5%, 98.3%; enrofloxacin 28.1%, 49.2%, 59.2%; florfenicol 11.3%, growth, 68.7%, tildipirosin 20.7%, 1.8%, 72.9%; and tulathromycin 14.2%, 66.3%, 44.5%. Ceftiofur (1.8 log_10_, 98.3% kill) killed more bacteria than did enrofloxacin (0.4 log_10_, 59.2% kill, *p* < 0.0001), pradofloxacin (0.6 log_10_, 70.7% kill, *p* < 0.0001), tildipirosin (0.7 log_10_, 72.9% kill, *p* = 0.0226) or tulathromycin (0.3 log_10_, 44.5% kill, *p* = 0.0003).

A statistically significant difference was seen for time (*p* < 0.0001) and treatment by time (*p* < 0.0001).

## 4. Discussion

Bacterial pathogens remain important infectious agents in BRD. The pathogenesis of BRD is influenced by a complex interaction between host factors, numerous environmental factors and bacterial and viral pathogens [22]. Stress is an important contributor to BRD. Viral pathogens, such as bovine herpes virus 1, parainfluenza-3 virus and bovine and respiratory syncytial viruses, serve to weaken the host defenses, facilitating lower respiratory tract colonization with bacteria. Animal transport, crowding, commingling along with weaning, dust, unfavorable weather and poor ventilation predispose the animals to secondary bacterial infections. While *P. multocida*, *Histophilus somni* and *Mycoplasma bovis* are associated with BRD, according to Griffon and colleagues, *M. haemolytica* is both the most common and the most serious of the bacterial agents [23]. Klima et al. reported on viral and bacterial pathogens in 68 BRD mortalities in Canada (Alberta) and the USA (Texas, Nebraska) [7]. *M. haemolytica* was detected in 91% and *P. multocida* in 13% of animals. As such, investigating these two bacterial pathogens in kill assays as described in this manuscript and against a new antimicrobial agent is relevant and important in understanding the potential uses for pradofloxacin for treatment of BRD.

The choice of an initial antimicrobial agent for therapy for respiratory tract infection could influence outcome. Bai and co-investigators compared the effectiveness of first-line and alternative antibiotics in human patients hospitalized with non-severe community-acquired pneumonia [24]. It was a retrospective study conducted from 2015 to 2021 with data from 19 Canadian hospitals. There were 23,512 patients, of which 9340 (39.7%) received a beta-lactam plus macrolide antibiotic combination regimen, 9146 (38.9%) received beta-lactam alone, 4510 (19.2%) received fluoroquinolone alone and 516 (2.2%) received a beta-lactam plus doxycycline regimen. Length of in-hospital stay (until discharge or death) was shorter for patients receiving beta-lactam/macrolide or fluoroquinolone (4.6 days for either regimen) versus 5.6 days for beta-lactam alone and 6 days for a beta-lactam/doxycycline regimen. The authors also indicated that their findings cannot exclude a clinically important increase in mortality for the beta-lactam-alone regimen and suggest this is in support of the beta-lactam, while an antibiotic alone was not recommended as a first-line regimen in the current pneumonia guidelines [25].

BRD is significant and estimated to cost the North American feedlot cattle industry more than USD 3 billion dollars annually [26]. BRD accounts for 70.8% of morbidity and 40–50% of mortality [27]. Investigating antimicrobial agents in kill assays utilizing varying densities of bacteria as reported here is also relevant, as bacterial densities are reported to fluctuate during infections [8]. Whereas MIC testing utilizes a bacterial inoculum of 10^5^ cfu/mL, MPC testing is based on ≥10^9^ cfu applied to the surface of drug-containing agar plates. High bacterial densities are reported from infections of the central nervous system [28], urinary tract [29] and lung [10,30,31]. Lebastard and colleagues reported on high-density bacterial burdens in bronchoalveolar lavage (BAL) fluid from dogs with lower respiratory tract signs [32]. Drusano et al. reported on a dilution factor of quantitative bacterial cultures of BAL specimens from human patients with ventilator-associated bacterial pneumonia [33]. Interestingly, quantitative cultures from BAL specimens indicated a bacterial burden of ≥10^4^ cfu/mL; however, considering the dilutional factor, 50% of patients had bacterial burdens that exceeded the inverse of the frequency of mutation to resistance—a density in the range of 1 × 10^7^ to 1 × 10^8^ cfu [34]. Drusano et al. reported on granulocytes being a major line of defense in pneumonia [35,36]. High bacterial burdens may saturate granulocytes, thereby overwhelming their function and suppressing their ability to kill the infecting organism. Drug therapy reducing bacterial density reduced the saturation of granulocytes, allowing these cells to further add >log_10_ (cfu/g) kill over 24 h. Granulocyte function appears maximal when the bacterial burden is reduced to <5 log_10_ (cfu/g). The authors further suggest that initial antimicrobial therapy should generate a minimum kill of 1.5–2.0 log_10_ (cfu/mL). In an excellent review by Drusano and colleagues on suppression of emergence of resistant-bacteria-infected mice that were dosed to give specific AUC/MIC ratios, an AUC/MIC ratio of 52 allowed for expansion of resistant populations, whereas a ratio of AUC/MIC of 157 suppressed mutant subpopulation amplification [37]. These measurements were conducted over 48 h versus the usual 24 h for such studies. Drusano et al. further commented on Monte Carlo simulations with levofloxacin (750 mg daily) and target attainment (AUC/MIC of 157) with MIC values of 0.5 µg/mL. Target attainment was slightly in excess of 80%, but across a range of MIC values one might readily expect in a clinical setting, exposure for resistance suppression would be approximately 62%, leading the authors to suggest that single-agent therapies might be difficult to attain resistance suppression with and that combination therapy might be more successful. How these observations apply to bug/drug combinations where AUC/MIC ratios are >157 or where dual-targeting drugs (such as pradofloxacin) are used requires further investigation. The drivers for resistance prevention may not always be the same (AUC/MIC, C_max_/MIC, Time > MIC) depending on microorganism and antimicrobial. For moxifloxacin—an antibiotic closely related to pradofloxacin—the C_max_/MIC ratio was important for resistance prevention with *Yersinia pestis* [38].

What drug concentrations should be used in kill assays as described in this manuscript? We used four separate drug concentrations, namely, the MIC, MPC, maximum serum and maximum tissue drug concentrations. The first two are reproducible measurements, and for serum or plasma concentrations, multiple measurements are taken over defined times. Mouton and colleagues commented on this in their article “Tissue concentrations: do we ever learn?” [39]. Potential problems with tissue drug concentration measurements have been previously highlighted and include the following: (1) there is a potential of intracellular and extracellular drug concentrations mixing when grinding tissues; (2) depending on the drug class, overestimation or underestimation of drug levels may occur depending on how the samples are collected; and (3) methodological problems may not be immediately apparent and may be variable between labs measuring such levels of individual drugs. Apley, commenting on pharmacodynamics, indicated it was imprecise in guiding therapy due to consideration of bound versus unbound drug, tissue versus plasma concentrations, drug degradation over time, microorganism variability and site of infection-specific variables [40]. Nielsen and Friberg in a comprehensive review on PK/PD modeling of antibacterial drugs indicated models that characterize bacterial growth, killing of bacteria by antibiotics and immunity and selection of resistance; their review may provide valuable information that may be used in PK/PD models [41]. However, few such models describe time courses of antibacterial drug effects in animals or patients. Such potential limitations as highlighted above always need to be considered when reviewing and evaluating data from in vitro studies, including this study.

In this study, pradofloxacin was bactericidal against *M. haemolytica* and *P. multocida* strains. In a recent publication from our laboratory, we reported for pradofloxacin a C_max_/MIC ratio of 212.5 and AUC/MIC_90_ of 825 based on testing 34 *M. haemolytica* strains and using published pharmacological data on pradofloxacin drug concentration; for *P. multocida* (n = 41), those values were 212.5 and 825, respectively. In considering a protein-binding percentage of 33%, the C_max_/MIC_90_ and AUC/MIC_90_ ratios would exceed the model estimates (AUC/MIC of 157) for suppressing mutant subpopulation amplification identified above.

We previously published on the comparative in vitro killing of the same isolates of *M. haemolytica* and *P. multocida* using eight of the nine antibiotics here and using a bacterial density of 10^5^ (100,000) cfu/mL. Additionally, the same drug concentrations were used [16]. In this study, the bacterial densities investigated ranged from 10^6^ (1 million) cfu/mL to 10^9^ (1 billion) cfu/mL—densities associated with infection.

In this study, pradofloxacin (and the other quinolones tested) were bactericidal. Against *M. haemolytica* using the MIC drug concentration, pradofloxacin killed between 80.3 and 98.4% of cells over the range of bacterial densities (10^6^–10^9^ cfu/mL) after 24 h of drug exposure; between 98.9 to 100% at the MPC drug concentration; between 99.9 to 100% at the C_max_ drug concentration following 30 min to 1 h of drug exposure; and between 92.1 an d100% at the maximum tissue drug concentration following 30 min to 1 h of drug exposure. For the *P. multocida* strains tested, pradofloxacin killed 69.5–96.2% of cells after 12 h of exposure to the MIC drug concentration; 70.3–99.7% of cells following 24 h of drug exposure to the MPC drug concentration; 84.4–94.4% following 4 h of drug exposure at the maximum serum drug concentration; and 73.4–96.2% following 2 h at the maximum tissue drug concentration. The results reported here are consistent with kill rate data recently reported by us on the same eight drugs and organisms but tested against 10^5^ cfu/mL in a 180 min kill rate assay [16].

Testing antibiotics against the range of bacterial densities reported here is relevant. As previously indicated, spontaneous mutation may occur over a range of bacterial densities between 10^7^ and 10^9^ cfu. Testing antimicrobials at ≥10^9^ cfu/mL, poor kill rates and/or organism regrowth occurred for some of the agents and drug concentrations tested. Whether this relates to mutant subpopulations amplifying in the presence of the drug or some other variable remains unknown, as end of kill assay investigations of the remaining bacterial populations were not performed. This could be the bases for further investigations. Mutant selection in the presence of pradofloxacin over the bacterial densities tested would be unexpected, as an organism would need to possess two simultaneous mutations for growth in the presence of the drug [42]. As a dual-targeting drug, resistance selection in the presence of pradofloxacin is expected to be a rare event [43]. As resistance to quinolones by mutation occurs in a step-wise fashion, a single mutation would be sufficient to raise the MIC to the drug, but two mutations are generally necessary to raise the MIC beyond the resistance breakpoint.

There are some limitations for this and other in vitro studies measuring antimicrobial activity: (1) In vitro susceptibility or resistance does not correlate 100% with clinical outcome. (2) All drug concentrations used in the kill assays in this study are single-drug concentrations representing a minimum (MIC) or maximum (i.e., C_max_) concentration, and the effect seen could be different from a total drug exposure, which is difficult to test in this model. Additionally, different drug concentration values than tested here might yield different results. (3) In vitro measurements use defined media, which is not a direct comparison with blood or epithelial lining fluid in vivo [44], and nor do in vitro measurements parallel drug diffusion or immune cell interactions that occur during infection. To this point, Buyck et al. compared MIC results for macrolides/ketolides tested against an American Type Culture Collection strain of *Pseudomonas aeruginosa* in different media [45]. MIC values for macrolides against *P. aeruginosa* are typically high (≥128 µg/mL) when tested by using standardized Mueller–Hinton Broth. Testing in Roswell Park Memorial Institute 1640 media yielded substantially lower MIC values (e.g., 1–16 µg/mL). Media can profoundly influence in vitro testing results. (4) Total drug concentrations for each drug were used and not adjusted for protein binding. (5) Our study did not evaluate declining drug concentrations over time and impact on bacterial killing. Mouton and Vinks reported that as drug concentrations decline, bacterial killing will decrease and a point at which the bacteria will not grow or are killed is called the stationary concentration [46]. The stationary concentration was close to the MIC for beta-lactam drugs, and for quinolones and aminoglycosides the stationary concentration was usually lower than the MIC. The authors concluded that the MIC is not a good PD parameter to characterize the concentration-effect relationship of a given antimicrobial. In our study, we found that MIC drug concentrations were variable in the rate and extent of bacterial killing for all drugs tested and over the densities investigated. Regardless of these limitations, this study allowed a comparison between the antimicrobial agents studied in a controlled standardized assay where differences and similarities between the agents can be measured. The statistically significant differences seen between antimicrobial agents is based on differences in colony count reduction in the presence of each drug and following timed drug exposure. How these in vitro observations of statistically significant differences between drugs manifest clinically requires further investigation.

Discriminate use of antimicrobial agents is essential, and concerns over environmental contamination by antibiotics and drug-resistant bacteria have been reviewed by Haenni et al. [47] and Das et al. [48]. As such, antibiotics should only be used when clinically indicated, and ongoing strategies for dealing with environmental concerns require more research and resource allocation.

## 5. Conclusions

In this study we compared the killing of bovine isolates of *M. haemolytica* and *P. multocida* by pradofloxacin and several comparative antimicrobial agents over a range of bacterial densities. Pradofloxacin killed as many or more bacterial cells than did other antimicrobial agents, and this was notable against the higher-density populations where less susceptible subpopulations may be present. Additionally, pradofloxacin was rapidly bactericidal over the drug concentrations tested. As a dual-targeting antibiotic, pradofloxacin has a low likelihood for resistance selection from fully susceptible bacteria based on low MPC values at or below the susceptibility breakpoint. This study represents the most comprehensive comparative killing dataset currently available and the first such data with bovine bacterial pathogens for pradofloxacin and tildipirosin. Pradofloxacin should be a welcome addition to the antimicrobials available for treatment of BRD.

## Figures and Tables

**Table 1:** Comparative MIC, MPC and therapeutic drug concentration values for 9 antimicrobial agents.

	Isolates						C_max_ (µg/mL)	T_issuemax_ (µg/mL)
	#13		#17		#36			
	MIC	MPC	MIC	MPC	MIC	MPC		
***M. haemolytica***								
Ceftiofur	0.031	0.063	0.008	0.125	0.008	0.125	6.9	2.64
Danofloxacin	0.016	0.125	0.016	0.125	0.016	0.25	1.69	6.25
Enrofloxacin	0.016	0.125	0.016	0.125	0.5	0.063	1.9	4.6
Florfenicol	2	4	2	2	0.031	2	4.5	2.94
Marbofloxacin	0.106	0.063	0.016	0.063	0.016	0.063	1.5	NT
Pradofloxacin	0.008	0.031	0.016	0.031	0.016	0.031	2.64	0.81
Tildipirosin	1	2	1	2	0.5	2	0.767	14.77
Tilmicosin	4	≥32	0.5	16	0.5	4	0.25	NT
Tulathromycin	1	8	0.5	2	0.5	2	0.6	3.2
***P. multocida***	**#5**		**#6**		**#14**		**C_max_** **(µg/mL)**	**T_issuemax_ (µg/mL)**
Ceftiofur	0.002	0.125	0.002	0.125	0.002	0.25	6.9	2.64
Danofloxacin	0.016	4	1	8	1	8	1.69	6.25
Enrofloxacin	0.008	0.063	0.004	0.063	0.008	0.031	1.9	4.6
Florfenicol	0.5	1	0.25	1	0.5	0.5	4.5	2.94
Marbofloxacin	0.016	0.125	0.008	0.125	0.016	0.25	1.5	NT
Pradofloxacin	≤0.008	0.031	0.031	0.031	0.004	0.25	2.64	0.81
Tildipirosin	1	4	0.5	4	0.5	4	0.767	14.77
Tilmicosin	4	32	2	8	2	4	0.25	NT
Tulathromycin	0.5	2	0.25	1	0.5	1	0.6	3.2

MIC = minimum inhibitory concentration; MPC = mutant prevention concentration; C_max_ = maximum serum drug concentration; T_issuemax_ = maximum tissue drug concentration; µg/mL = microgram per milliliter; NT = not tested.

## Data Availability

The raw data supporting the conclusions of this article will be made available by the authors on request.
